# Crystal Structure of the Cohesin Gatekeeper Pds5 and in Complex with Kleisin Scc1

**DOI:** 10.1016/j.celrep.2016.02.020

**Published:** 2016-02-25

**Authors:** Byung-Gil Lee, Maurici B. Roig, Marijke Jansma, Naomi Petela, Jean Metson, Kim Nasmyth, Jan Löwe

**Affiliations:** 1 MRC Laboratory of Molecular Biology, Cambridge CB2 0QH, UK; 2 Department of Biochemistry, University of Oxford, Oxford OX1 3QU, UK

**Keywords:** sister chromatid cohesion, Smc proteins, Scc3

## Abstract

Sister chromatid cohesion is mediated by cohesin, whose Smc1, Smc3, and kleisin (Scc1) subunits form a ring structure that entraps sister DNAs. The ring is opened either by separase, which cleaves Scc1 during anaphase, or by a releasing activity involving Wapl, Scc3, and Pds5, which bind to Scc1 and open its interface with Smc3. We present crystal structures of Pds5 from the yeast *L. thermotolerans* in the presence and absence of the conserved Scc1 region that interacts with Pds5. Scc1 binds along the spine of the Pds5 HEAT repeat fold and is wedged between the spine and C-terminal hook of Pds5. We have isolated mutants that confirm the observed binding mode of Scc1 and verified their effect on cohesin by immunoprecipitation and calibrated ChIP-seq. The Pds5 structure also reveals architectural similarities to Scc3, the other large HEAT repeat protein of cohesin and, most likely, Scc2.

## Highlights

• The crystal structure of the cohesin subunit Pds5 was determined
• The crystal structure of Pds5 in complex with Scc1 binding region was determined
• Structure-based mutants in Pds5 and Scc1 were analyzed by coIP and ChIP-seq
• Pds5 shows some similarity to Scc3, the other large HEAT repeat cohesin subunit

## Graphical Abstract

**Figure:**
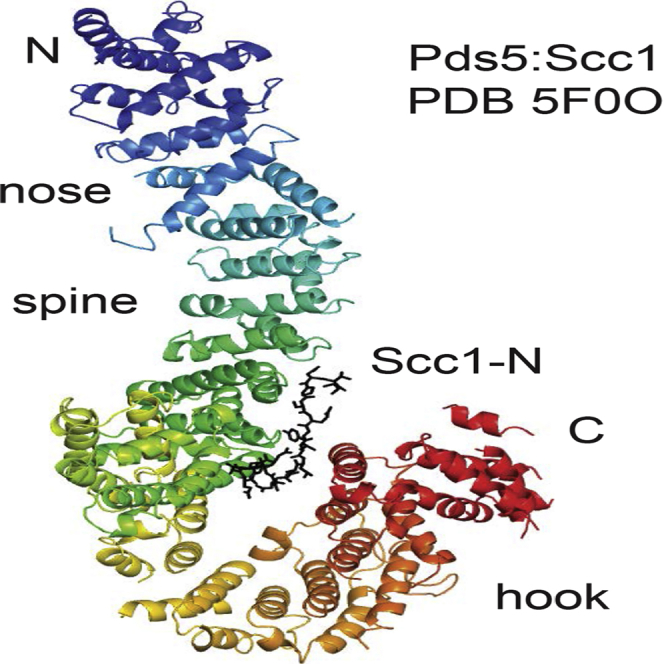

## Teaser

Cohesin entraps DNA in a ring, facilitating sister chromatid cohesion during mitosis and meiosis. Lee et al. describe the structure of the cohesin subunit Pds5 in complex with Scc1, cohesin’s kleisin, mutants disrupting the Pds5:Scc1 interaction, and structural similarity to another cohesin subunit, Scc3.

## Introduction

The segregation of multiple chromosomes during mitosis in eukaryotes is made possible by sister chromatid cohesion whose destruction triggers the simultaneous disjunction of all sister chromatid pairs at the metaphase-to-anaphase transition. Cohesion is mediated by the cohesin complex, at whose core is a heterodimer of coiled-coil Smc1 and Smc3 proteins (structural maintenance of chromosomes), each with a hinge dimerization domain at one end and an ABC ATPase head domain at the other (Nasmyth and Haering, 2009, Onn et al., 2008). The latter are bridged by the alpha kleisin Scc1, forming a molecular ring up to 50 nm in contour length, within which sister DNA can be entrapped (Haering et al., 2002, Haering et al., 2008). Anaphase is triggered through cleavage of Scc1 by the protease separase, whose activity is regulated through the cell-cycle-specific destruction of an inhibitor securin by the anaphase-promoting complex/cyclosome (APC/C) (Uhlmann et al., 1999). Scc1 cleavage opens the cohesin ring, permitting traction of sister chromatids to opposite poles through pulling forces associated with microtubules attached to kinetochores. Loading of cohesin onto chromosomes, as well as separase-independent unloading (release), depends on several other proteins, Scc3, Pds5, Wapl, and Scc2/4, that interact with the core ring subunits (Ciosk et al., 2000, Hartman et al., 2000, Kueng et al., 2006, Losada et al., 2000, Panizza et al., 2000, Rankin et al., 2005, Schmitz et al., 2007, Tóth et al., 1999).

Cohesin loading requires ATP hydrolysis, Scc3, and Scc2/4 (Arumugam et al., 2003, Arumugam et al., 2006, Ciosk et al., 2000, Hu et al., 2011, Weitzer et al., 2003). Loading is believed to require opening of a gate created by transient dissociation of the Smc1/3 hinge interface (Gruber et al., 2006). Unloading occurs through two mechanisms: in addition to irreversible opening of the ring by separase, cohesin has a “releasing activity” that enables a dynamic association with chromosomes, in particular during G1 phase of the cell cycle (Gandhi et al., 2006, Kueng et al., 2006, Nishiyama et al., 2010). Though active throughout the cell cycle, releasing activity is most active, at least in animal cells, during mitosis, when it is responsible for removing a large fraction of cohesin from chromosome arms in the prophase pathway (Losada et al., 1998, Waizenegger et al., 2000). Centromeric cohesin is protected from the prophase pathway by shugoshin-mediated recruitment of PP2A to centromeres (Riedel et al., 2006, Tang et al., 2006).

The observation that releasing activity is abolished by fusing Scc1’s N terminus to Smc3’s C terminus suggests that release occurs through the escape of DNA from the ring through a gate between Scc1’s N-terminal domain and Smc3’s coiled coil (see Figure 2B for an overview) (Chan et al., 2012). For releasing activity not to destroy all sister chromatid cohesion, it is countered by acetylation during S phase of a pair of lysine residues in Smc3’s ATPase head by the Eco1 acetyl transferase (Ivanov et al., 2002, Nishiyama et al., 2010, Rolef Ben-Shahar et al., 2008, Rowland et al., 2009, Unal et al., 2008).

Consistent with the notion that the main role of acetylation is to block release, mutants defective in releasing activity enable cells to proliferate in the absence of Eco1 (Rolef Ben-Shahar et al., 2008, Rowland et al., 2009, Sutani et al., 2009, Tanaka et al., 2001). Indeed, most releasing activity mutations, be they in Smc3, Pds5, Scc3, or Wapl, were initially identified as *eco1* suppressors. Smc3 deacetylation, which is mediated by Hos1 in yeast (Beckouët et al., 2010, Borges et al., 2010, Xiong et al., 2010) and HDAC8 in mammalian cells (Deardorff et al., 2012), takes place upon Scc1 cleavage at the onset of anaphase.

In addition to being crucial for the release of cohesin from chromosomes, during S phase, Pds5 promotes the acetylation of Smc3 that will protect cohesin from releasing activity in G2 (Chan et al., 2013, Vaur et al., 2012). It also prevents deacetylation by Hos1, from S phase until Scc1 cleavage at the onset of anaphase. Lastly, at least in yeast, Pds5 has a role in maintaining sister chromatid cohesion during G2/M by a mechanism that does not involve Smc3 acetylation (Chan et al., 2013, Tong and Skibbens, 2014). Thus, Pds5 can be considered the gatekeeper of the cohesin ring. A complementary study investigating the interaction of Pds5 and Scc1 and the structure of their complex is reported in this issue of *Cell Reports* (Muir et al., 2016).

## Results and Discussion

### Structure of *Lachancea thermotolerans* Pds5

**Figure 1:**
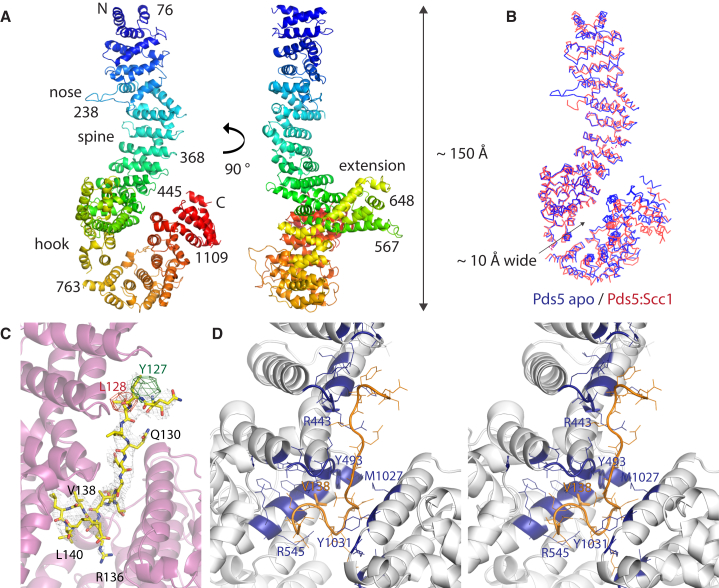
Crystal Structures of Pds5 from *L. thermotolerans* in the Apo Form and Bound to Scc1 (A) Crystal structure of Pds5 from *L. thermotolerans*. Residues are colored from the N terminus (blue) to the C terminus (red). Pds5 is composed of a large number of HEAT repeats, many of them irregular to produce protrusions, such as the nose and the extension domain, that consist of helices that are additions to the regular HEAT repeat. The N and C termini lie at opposite ends of the 150-Å-long molecule. The C-terminal hook bends back, creating a contact between the hook and the spine and forming a small ring. (B) Conformational changes between Pds5 in the apo form and when bound to a peptide containing Scc1’s Pds5-binding region. Because the Scc1 peptide is wedged between the hook and the spine, the hook slightly widens upon Scc1 binding. (C) Close-up of the 2Fo-Fc electron density map and the fitted Scc1 peptide that extends from residue 125 to 141. The orientation here is similar to that in (A) and (B). The densities shown in green and red are the result of two separate SeMet SAD experiments with Scc1 peptides that contained SeMet residues at the indicated positions Scc1(Y127SeMet) and Scc1(L128SeMet), revealing the sequence and direction of the fitted peptide. (D) Stereo view of the bound Scc1 peptide. Key binding residues in Pds5 are highlighted in blue. Most notably, M1027 and Y1031 on the hook (right) also mediate contact between the hook and spine (left) in Pds5 apo. On the spine, interacting residues include I403, R410, R443, E444, T445, R446, Y492, Y493, I494, N495, K535, S538, S539, A542, F543, and R545. We found two residues that compromised the viability of yeast strains when the corresponding residues were mutated: Scc1(V138) and Pds5(Y493) (Figure 2A). See also Figures S1–S3.

We expressed Pds5 from *Lachancea thermotolerans* (Lt) in *E. coli* and determined its structure at 3.2 Å resolution (Experimental Procedures; Figures 1A and S1). LtPds5 is 47% identical in sequence to *Saccharomyces cerevisiae* (Sc) Pds5. The large molecule is exclusively alpha helical, composed of a large number of HEAT-like repeats and helical extensions/additions that deviate from the HEAT repeat pattern. The HEAT repeat pattern leads to a linear path from the N terminus to the C terminus, separating them by more than 100 Å. Deviations from the HEAT repeat pattern create a nose and extension domain (Figure 1A), as well as a very pronounced hook, bending back so that the most C-terminal portion of Pds5 contacts the middle section, which contains the most regular HEAT repeats and which we called the spine. Bending back the hook creates a small loop or ring with an inner diameter of approximately 10 Å.

### Structure of Pds5 Bound to Scc1

The region within kleisin Scc1 of cohesin that binds Pds5 has been mapped previously (Chan et al., 2013), and we therefore synthesized a 23-amino-acid-long peptide from *L. thermotolerans* Scc1 (121–143) containing this region. Although binding was weak (Chan et al., 2013), molecular replacement and a difference Fourier map with data collected from co-crystals of LtPds5 containing the LtScc1 peptide revealed clear difference density. We could locate and model residues 125–141 of Scc1 in the density (Figure 1C; Table 1), using data from two SeMet single-wavelength anomalous diffraction (SAD) datasets from crystals with Scc1 peptides containing SeMet residues to confirm the sequence assignment (Figure 1C, red and green densities).

Molecular replacement with four fragments was required because Pds5 shows changes in conformation upon peptide binding (Figure 1B). The hook opens and the entire structure bends such that the most N-terminal part (Figure 1B, left, top) moves by up to 10 Å relative to the central part (superposition based on Cα atoms of residues 473 to 726). The nose becomes disordered in the co-crystals.

**Figure 2:**
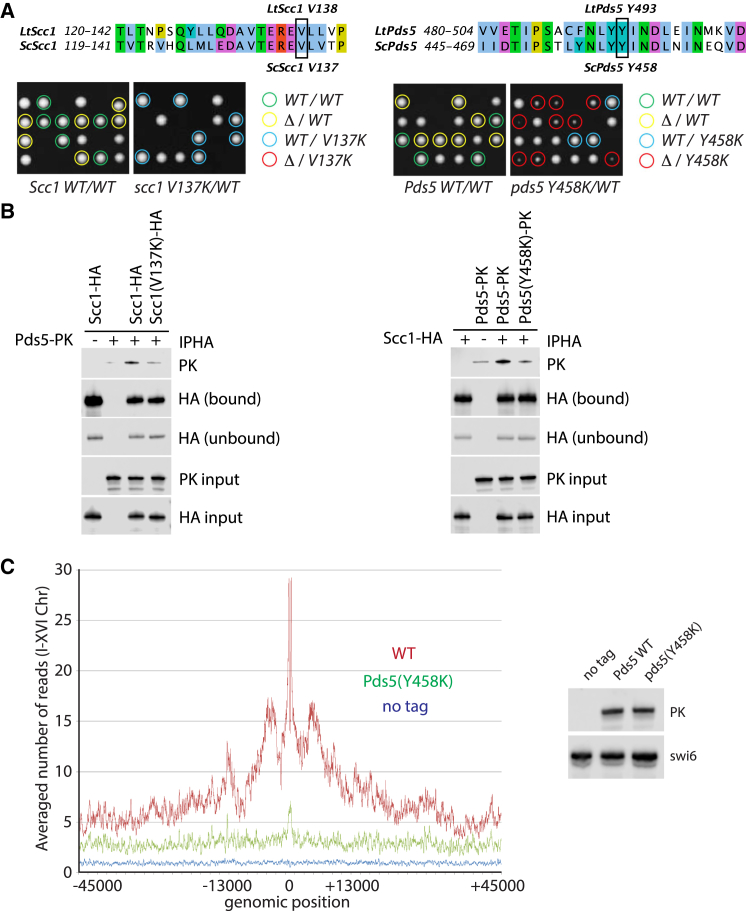
Analysis and Validation of the Pds5:Scc1 Interaction in *S. cerevisiae* (A) Validation of the Pds5:Scc1 complex through yeast mutant viability analysis in *S. cerevisiae*: tetrad dissection of *Sc*Scc1(V137K) and *Sc*Pds5(Y458K). Sequence alignments indicate equivalent residues in *L. thermotolerans* and *S. cerevisiae*. Left: heterozygous diploids with one endogenous *SCC1* locus deleted (strain K12714) carrying either wild-type (K25166) or mutant *scc1 V137K* (K24958) genes integrated at the *leu2* locus were sporulated on YPD plates and four-spored asci dissected. Right: heterozygous diploids with one endogenous *PDS5* locus deleted (K25105) carrying either wild-type (K25106) or mutant *pds5 Y458K* (K25108) genes integrated at the *lys2* locus were sporulated on YPD plates and four-spored asci dissected. The resultant genotypes are color-coded. Note that strains expressing just scc1(V137K) or pds5(Y458K) (K25126) are lethal or sick, respectively, but neither of these mutations cause a dominant-negative effect when co-expressed with Scc1 WT (K25002) or Pds5 WT (K25120). (B) Immunoprecipitation of Scc1 and detection of the co-precipitated Pds5 showing that scc1(V137K) (K24595, K25118, K25202, and K25206) and pds5(Y458K) (strains K24593, K25120, K25204, and K25210) greatly reduce the interaction with Pds5 and Scc1, respectively. (C) Calibrated ChIP-seq profiles of Pds5 (strain K25120) and pds5(Y458K) (K25128) showing the number of reads at each base pair away from the CDEIII element averaged over all 16 chromosomes. Right: demonstration of equal Pds5 protein levels in those strains by western blotting. A non-averaged profile, a difference plot, and fluorescence-activated cell sorting data showing cycling cells are shown in Figures S5A–S5C. See also Figures S4 and S5.

**Figure 3:**
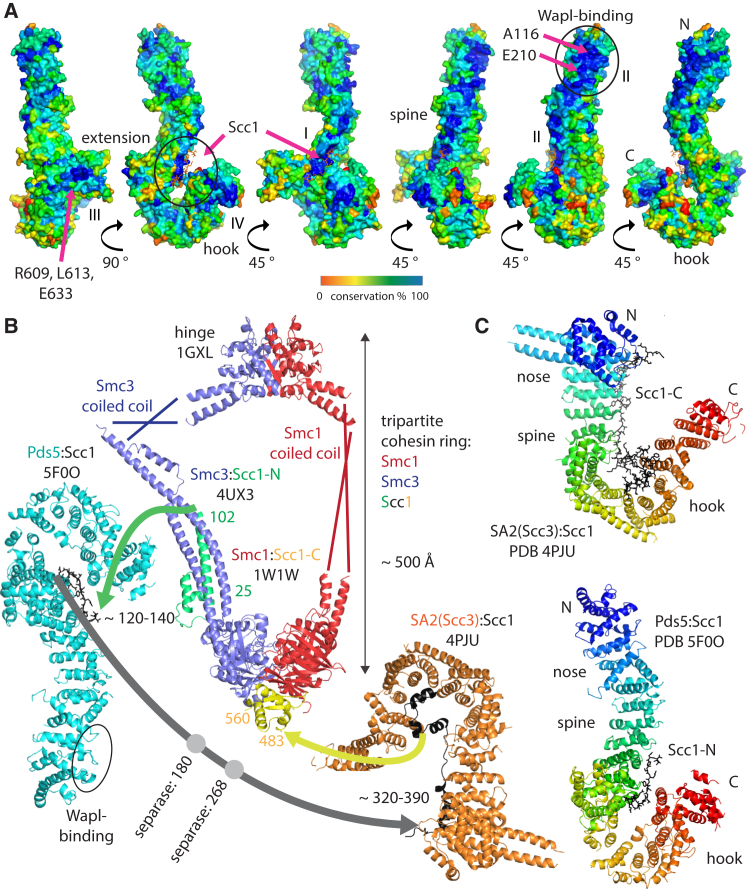
The Pds5 Structure in the Context of the Cohesin Complex (A) Sequence conservation mapped onto the molecular surface of Pds5 in the Scc1-bound form. The Scc1 peptide is highlighted with arrows and is shown in orange. Four major areas of strong conservation have been detected: the Scc1 binding site on the spine and hook, validating Scc1’s binding site on Pds5 (I). Extending toward the N terminus upward, conservation runs all the way from region I along the spine to the N-terminal domain, constituting region II. Previous mutants in Pds5 that were shown to reduce Wapl recruitment (Rowland et al., 2009) indicate that Wapl likely binds to the N-terminal region of Pds5 as indicated. Two additional patches are on the back of the extension domain (III) and at the tip of the hook (IV). Relative rotations are indicated by arrows, the panel on the left corresponds to the right panel in Figure 1A. (B) To-scale exploded view drawing of the cohesion complex, including structurally known parts. The basic tripartite ring is made out of Smc1 (red) (Haering et al., 2004) and Smc3 (blue) (Gligoris et al., 2014) that dimerize through their hinge domains (top) (Haering et al., 2002). The Smc coiled-coil regions without crystal structures are indicated by lines and are not to scale. Kleisin Scc1 bridges the Smc ATPase head domains that are forming a heterodimer primed for ATP hydrolysis. The Smc3 head binds Scc1’s N-terminal domain (green). Smc1 head domain binds the C-terminal domain of Scc1 (yellow). Residues in the middle of Scc1 are indicated by arrows as they have not been resolved by crystal structures, including the protease sites for separase. N-terminal to those separase sites, very close to the Scc1 site that binds the Smc3 head, lies the Scc1 site that binds between the hook and spine of Pds5. C-terminal to the separase sites is the site that binds between the hook and spine of Scc3 (Hara et al., 2014, Roig et al., 2014). Given that both Pds5 and Scc3 exhibit strong sequence conservation outside the known Scc1 binding surfaces, it is likely that these represent binding sites for Wapl and possibly sites on the Smc proteins. The Scc2/4 loading complex also has to interact with cohesin as loading proceeds through the hinge domains. Separase-independent unloading (releasing activity) most likely occurs through opening of the Smc3:Scc1-N interface, and it is therefore not surprising given the shown relative approximate positions that Pds5 has been implicated in the releasing activity. Note that the exact orientations of Pds5 and Scc1 with respect to the tripartite ring are not known and the drawing is not the result of docking calculations. (C) Scc3/SA and Pds5 share overall architecture, including Scc1 binding. Both proteins are part of the cohesin complex and bind to cohesin’s kleisin. Scc3 and Pds5 are large, irregular HEAT repeat proteins that separate N and C termini by large distances. Scc1 binding occurs mostly between the hook and spine in both proteins, creating a smaller ring in Scc3 than in Pds5. The extension and nose are less well conserved but still discernable. The nose is disordered in the Scc1-bound crystal form of Pds5 but visible in the apo form (Figure 1A, left). See also Figure S6 for subdomains of Pds5 aligned against Scc3/SA2. See also Figure S6.

In the Pds5:Scc1 complex structure, the Pds5 hook opens because the Scc1 peptide is wedged between the spine and the end of the hook, maintaining a closed-ring architecture (Figure 3C, bottom). Acidic and hydrophobic residues that in the apo structure of Pds5 make the contact with the spine, D999, M1027 and Y1031, now interact with the Scc1 peptide (Figure 1D). On the Pds5 spine many Pds5 amino acid side chains are in contact with the Scc1 peptide, as shown and listed in Figure 1D. Substitution by lysine of the residue equivalent to LtY493 in *S. cerevisiae*, namely, Y458K, greatly reduced proliferation and caused temperature-sensitive lethality (Figures 2A, right, and S4). In contrast, mutating the hydrophobic residues M1027 and Y1031 (located on the hook, I998 and F1002 in Sc) to lysine did not lead to lethality.

In the complex, Scc1 is in a mostly extended conformation, except toward its C terminus, which forms a more compact arrangement with helical turns. We found that V138 mutated to lysine was lethal in *S. cerevisiae* ScScc1(V137K) (Figure 2A, left), confirming previous results (Chan et al., 2013). In contrast, other mutations, namely, ScScc1 L126K, L128E, V132K, T133K, E134K, and E136K, had little or no effect (corresponding to Lt L128K, L129E, V133K, T134K, E135K, and E137K). As is indicated in Figure 1D, Scc1 V138 sits in a deep pocket in Pds5, lined by Pds5 Y493 and other hydrophobic residues.

To verify that the mutations function through a specific effect on the cohesin complex in cells, we performed immunoprecipitations of labeled cohesin subunits, expressed from endogenous promoters with and without the mutations ScScc1(V137K) and ScPds5(Y458K), determining the amounts of co-precipitated Pds5 and Scc1 by western blotting (Figure 2B). In both cases, a marked reduction was detected, more so with the Scc1 mutation. Calibrated chromatin immunoprecipitation sequencing (ChIP-seq) (Hu et al., 2015) showed that the ScPds5(Y458K) mutant greatly reduced Pds5’s association with chromosomal cohesin, especially in pericentric sequences (Figure 2C). We showed previously that this is not due to a defect in cohesin loading onto chromosomes since Scc1(V137K), defective in Pds5 recruitment, does not affect loading (Chan et al., 2013).

We conclude that both Scc1(V138) and Pds5(Y493) are required for the Pds5:Scc1 interaction and that our structure reflects this interaction well.

### Other Pds5 Interaction Regions

When we plotted sequence conservation among Pds5 homologs onto the Pds5 structure, several regions of potential functional interest became apparent (Figure 3A). The Scc1 peptide binding surfaces on Pds5, both on the hook and on the spine are well conserved, as expected for a binding site (region I). Extending along one edge of the spine, region II runs all the way to the N-terminal region at the top, where it ends with a large patch of conservation. This region includes a loop with consensus sequence APDAP (residues 116–120 in LtPds5). Regions III and IV are located on the extension domain and hook respectively. Region III is highly conserved in fungi, but not, apparently, in plants and animals, while region IV is conserved in all eukaryotes. As expected from such a large protein, it is likely that these regions correspond to the various interactions Pds5 makes with other cohesin subunits.

*S. cerevisiae* Pds5 mutations that suppress the lethality of *eco1* mutants and are therefore defective in releasing activity cluster in two domains (Rowland et al., 2009): the first cluster is found in and around the conserved APDAP loop (116–120 LtPds5 numbering), as well as the nearby and conserved glutamate E181 (Lt: E210). Mutations in this region either abolish (A88P, Lt: A116) or reduce (E181K, Lt: E210) association of Wapl with chromosomal cohesin in vivo (Chan et al., 2012) and are therefore implicated in binding Wapl (Figure 3A, region II, N-terminal region). The second cluster of *eco1* lethality suppressors is found within the (conserved within fungi) R578, L582, and E602 (Lt: R609, L613, and E633) patch on one side of the extension domain (Figure 3A, region III) (Rowland et al., 2009). Mutations here do not seem to affect Wapl recruitment (Chan et al., 2012) and must affect some other aspect of releasing activity. Pds5 must therefore have a role in releasing activity beyond merely recruiting Wapl.

### Implications for Releasing Activity

The Scc1 region that is shown here (Figures 1C and 1D) to bind directly to Pds5 contains a previously unreported motif, conserved in fungi, animals, and plants (Figure S2), which is only 10 to 20 amino acids downstream of the part of Scc1 that has been shown to bind to the coiled-coil segment of Smc3 (Figure 3B, green domain and green arrow) (Gligoris et al., 2014). This observation has two implications: first, the mode by which Scc1 binds Pds5 as elucidated here will prove similar in other eukaryotes. Second, Pds5 may be positioned such that its N-terminal region could lie close to the Smc3’s ATPase head and possibly Smc3’s K112 and K113, whose acetylation is so crucial for releasing activity. Recent crosslinking experiments support the idea of contacts between Pds5 and Smc3 head and coiled-coil domains (Huis in ’t Veld et al., 2014).

### Potential Structural Similarity of Pds5, Scc3, and Scc2

When comparing the structure of Pds5 with the other HEAT-repeat-containing subunit of cohesin, Scc3 (Hara et al., 2014, Roig et al., 2014), striking similarities appear: both proteins contain hook, spine, extension, and nose and, intriguingly, bind their corresponding conserved Scc1 sites (Figures S2 and S3) between the spine and the hook, forming a closed ring, and possibly creating another case of topological entrapment in the system (Figure 3C). Furthermore, both Pds5 and Scc3 have similar overall dimensions and separate the N- and C-terminal parts by a large distance. Because the precise amounts of bending at each HEAT repeat are different, overall structural alignments produce poor fits, although Pds5 subdomains containing the canonical HEAT repeat fold can be aligned reasonably well on their counterparts in Scc3/SA2 (Figure S6).

Furthermore, recent single-particle electron microscopy of the cohesin loader subunit Scc2, another HEAT-repeat-containing protein, shows overall architecture similar to that of Scc3 and Pds5 (Chao et al., 2015, Hinshaw et al., 2015). We speculate that these architectural similarities point toward shared mechanisms between Scc3 and Pds5 and, probably, Scc2.

Figure 3B depicts all structurally known cohesin subunits (except Wapl) to scale, showing the large sizes of Pds5 and Scc3 with respect to the Smc ATPase head domains and their attached coiled coils. It is clear that Scc1 plays a key role in the architecture of the complex as its path most likely orchestrates the positions of the various components.

The structures will provide the basis for determining which parts of Pds5 promote Smc acetylation, prevent Smc3 deacetylation, and help to maintain long-term cohesion. In the long-run they will help clarify the mechanism by which cohesin is released from chromosomes.

## Experimental Procedures

Full details are provided in the Supplemental Experimental Procedures.

*Lachancea thermotolerans* Pds5 (XP_002553028.1) was expressed as a C-terminal His_6_-tagged fusion in *Escherichia coli* using a T7 plasmid system and purified using metal affinity chromatography, anion exchange, and size-exclusion chromatography. Selenomethionine-labeled LtPds5 proteins were expressed using a published feedback inhibition procedure (van den Ent et al., 1999, Van Duyne et al., 1993) and purified using the same protocol for the native proteins. Scc1 peptide was chemically synthesized as were two otherwise identical, selenomethionine-substituted mutant peptides, Y127SeMet and L128SeMet. After crystallization, Se-Met SAD X-ray diffraction datasets were collected on beamlines i03 at Diamond Light Source and id23eh1 at the ESRF. The apo structure was determined by SAD using established procedures as implemented in the Crank2 pipeline (Skubák and Pannu, 2013). The Scc1 complex structure was determined by molecular replacement using fragments of the apo structure as search models. Modeling of the Scc1 sequence was guided by two additional selenium SAD experiments using peptides containing SeMet residues in two positions: LtScc1(Y127SeMet) and LtScc1(L128SeMet). For refinement, the datasets (Table 1) were corrected for anisotropy using the UCLA Diffraction Anisotropy Server (http://services.mbi.ucla.edu/anisoscale/) (Strong et al., 2006). Co-immunoprecipitations (coIPs) were performed using strains with epitope-tagged yeast strains, expressing proteins from endogenous promoters. IPs used HA epitope-directed commercial antibodies, and PK epitopes were also detected with commercial antibodies. Calibrated ChIP-seq was performed as described (Hu et al., 2015).

## Author Contributions

B.-G.L., M.B.R., N.P., K.N., and J.L. designed and conducted the experiments. B.-G.L. and M.J. performed protein sample preparation. B.-G.L. crystallized and collected X-ray datasets. B.-G.L. and J.L. determined the crystal structures. M.B.R. and J.M. performed mutant viability assays. M.B.R. performed coIP and ChIP-seq, and N.P. analyzed ChIP-seq. B.-G.L., K.N., and J.L. analyzed data and prepared the manuscript.

## Figures and Tables

**Table 1:** Crystallographic Data

Statistics			
Sample	*L. thermotolerans* Pds5 SeMet	*L. thermotolerans* Pds5 Native	*L. thermotolerans* Pds5:Scc1 Complex Native
NCBI Database IDs	XP_002553028.1	XP_002553028.1	XP_002553028.1, XP_002555756.1
Constructs	M-45-1221-LHHHHHH	M-45-1221-LHHHHHH	M-35-1221-LHHHHHH, Scc1 peptide 121-143: LTNPSQYLLQDAV TEREVLLVPG
**Data Collection**			
Beamline	Diamond I03	ESRF id23eh1	ESRF id23eh1
Wavelength (Å)	0.97941	0.97960	0.97949
Method	SeMet SAD	isomorphous to SeMet	molecular replacement
**Crystal**			
Space group	H3	H3	H3
Cell (Å)	237.5, 237.5, 80.5, 120°	238.2, 238.2, 80.7, 120°	235.7, 235.7, 94.4, 120°
**Scaling**			
Resolution (Å)	3.2	3.2	3.6
UCLA anisotropy (Å)^a^	na	3.2, 3.2, 3.5	3.5, 3.5, 4.5
Completeness (%)^b^	100.0 (100.0)	100.0 (100.0)	99.9 (99.9)
Multiplicity^b^	16.4 (16.5) merged two crystals	10.5 (10.8) one crystal	5.2 (5.4) one crystal
(I)/σ(I)^b^	15.5 (2.5)	14.5 (2.3)	11.2 (1.4)
R_merge_^b^	0.133 (1.385)	0.109 (1.141)	0.079 (1.188)
R_pim_^b^	0.050 (0.513)	0.056 (0.365)	0.060 (0.891)
CC1/2^b^	0.999 (0.884)	0.999 (0.871)	0.999 (0.682)
Anomalous correlation^b^	0.719 (0.048)		
Selenium sites	15 (100%)		
**Refinement**			
R/R_free_^c^		0.236/0.295	0.232/0.291
Model		76–278, 288–688, 692–726, 736–751, 763–1067, 1072–1109, 40 unsequenced residues at N and C termini, no waters	80–278, 287–688, 692–726, 736–751, 763–1067, 1072–1109, 40 e at N and C, Scc1 peptide 125–141 no waters
Bond length RMSD (Å)		0.003	0.005
Bond angle RMSD (°)		0.726	0.837
Favored (%)^d^		99.5	98.4
Disallowed (%)^d^		0.2	0.3
MOLPROBITY score		100^th^ percentile	99^th^ percentile
PDB IDs		5F0N	5F0O

a Correction for anisotropy applied through online server (http://services.mbi.ucla.edu/anisoscale/). Resolution limits along the a^∗^, b^∗^ and c^∗^ directions are listed.

b Values in parentheses refer to the highest recorded resolution shell.

c 5% of reflections were randomly selected before refinement and kept throughout all procedures.

d Percentage of residues in regions of the Ramachandran plot (PROCHECK).

## Accession Numbers

The accession numbers for the coordinates and structure factors reported in this paper have been uploaded to the Protein Data Bank: 5F0N (apo-LtPds5) and 5F0O (LtPds5:LtScc1 peptide complex).

## Supplemental Information

Document S1. Supplemental Experimental Procedures and Figures S1–S6

Document S2. Article plus Supplemental Information
