# Supplementary material for: Crystal Structure of the Cohesin Gatekeeper Pds5 and in Complex with Kleisin Scc1
Source: Cell Rep. 2016 Feb 25;14(9):2108–15. doi: 10.1016/j.celrep.2016.02.020 (PMC4793087; doi:10.1016/j.celrep.2016.02.020)
Supplement: Document S1. Supplemental Experimental Procedures and Figures S1–S6 [file mmc1.pdf]

Cell Reports, Volume *14*

Supplemental Information

Crystal Structure of the Cohesin Gatekeeper Pds5 and in Complex with Kleisin Scc1

Byung-Gil Lee, Maurici B. Roig, Marijke Jansma, Naomi Petela, Jean Metson, Kim Nasmyth, and Jan Löwe

Inventory of Supplemental Information

Supplemental Figure S1

Electron density map supporting the main finding of the paper, the crystal structure of Pds5 (belongs to Figure 1A).

Supplemental Figures S2 & S3

Multiple sequence alignments showing the conservation of Scc1 binding to Pds5 and Scc3 (belongs to Figure 1C).

Supplemental Figure S4

Lethality analysis of Pds5 and Scc1 mutants, validating the structure of the complex of Pds5 and Scc1 (belongs to Figure 2A &B).

Supplemental Figure S5

Control experiments supporting the ChIP-seq experiment in Figure 2C.

Supplemental Figure S6

Superposition of parts of the Pds5 and previous Scc3 structures, supporting Figure 3C.

Supplemental Experimental Procedures

List of Strains

Supplemental References

Supplementary Figure S1. 2Fo-Fc electron density map of apo Pds5 at 3.2 Å resolution. Related to Figure 1A. The map was contoured at 1.2 σ level. Inset: close up of a section of the spine, showing approximately residues 370-470. The electron density map was of excellent quality except the very N- and C-terminal sections, most likely through disorder in the crystals.

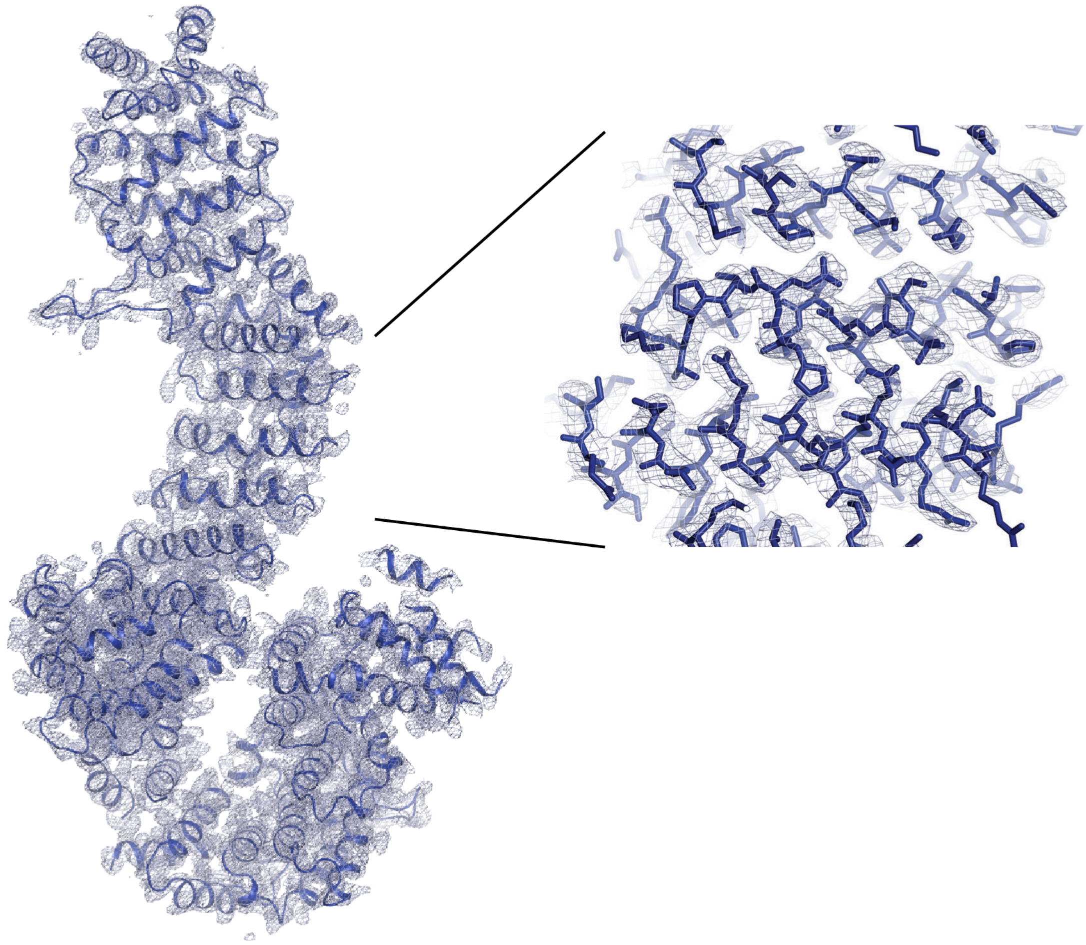

Supplementary Figure S2. Multiple sequence alignment showing conserved Pds5 binding regions within Scc1 kleisins. Related to Figure 1C. Up to 10 sequences similar to each *L. thermotolerans*, *S. cerevisiae*, *S. pombe*, *A. thaliana*, *H. sapiens* and *D. melanogaster* Scc1 were selected by BLAST (each block evenly sampled down to around 30 % sequence identity) and sequences were aligned by Clustal Omega. Resides 120-141 are shown for the Pds5 binding site in Scc1, containing more than the peptide co-crystallised here with Pds5 (*Lt* numbering; *Lt*Scc1 is the first sequence in the alignment.). LtPds5 residues 125-141 were built in the co-crystal structure (Table 1, Figure 1C, D).

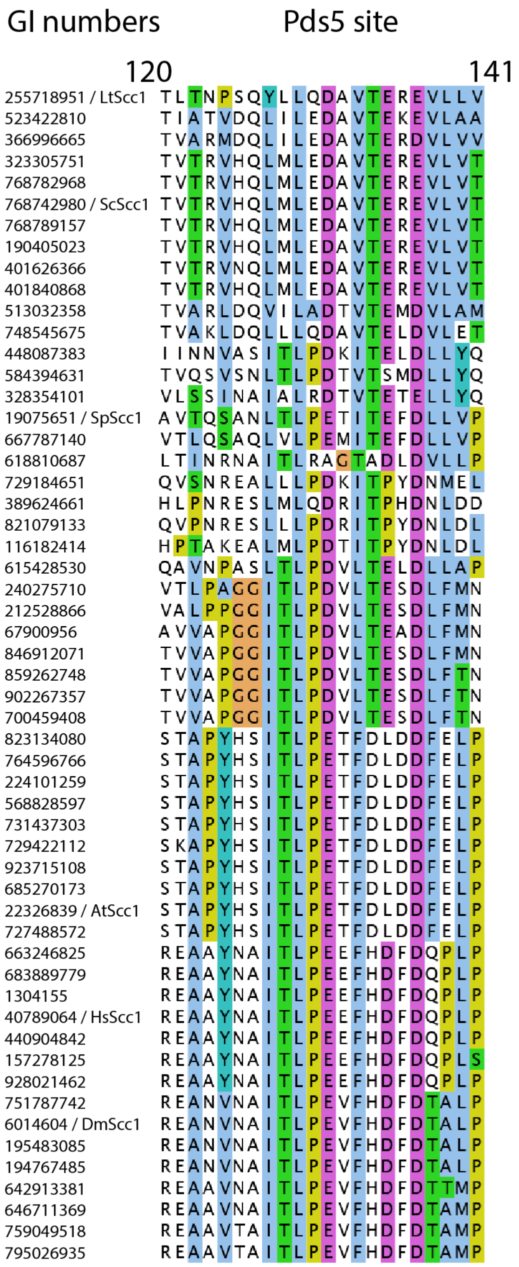

Supplementary Figure S3. Multiple sequence alignment showing conserved Scc3 binding regions within Scc1 kleisins from humans to yeast. Related to Figures 1C and 3C. 500 sequences similar to human and yeast Scc1 were selected by BLAST and all sequences (including from *L. thermotolerans*) were aligned by Clustal Omega. The alignment was truncated to 60 sequences without re-aligning. Resides 326-400 are shown for the Scc3 binding site in Scc1 corresponding to the fragment co-crystallised with SA2/Scc3 previously (*Lt* numbering; *Lt*Scc1 is the last sequence in the alignment.). Note that helix B is most likely missing from the Scc1 fragment in yeasts and related organisms but otherwise the alignment indicates that the binding mode of Scc1 to Scc3 is conserved.

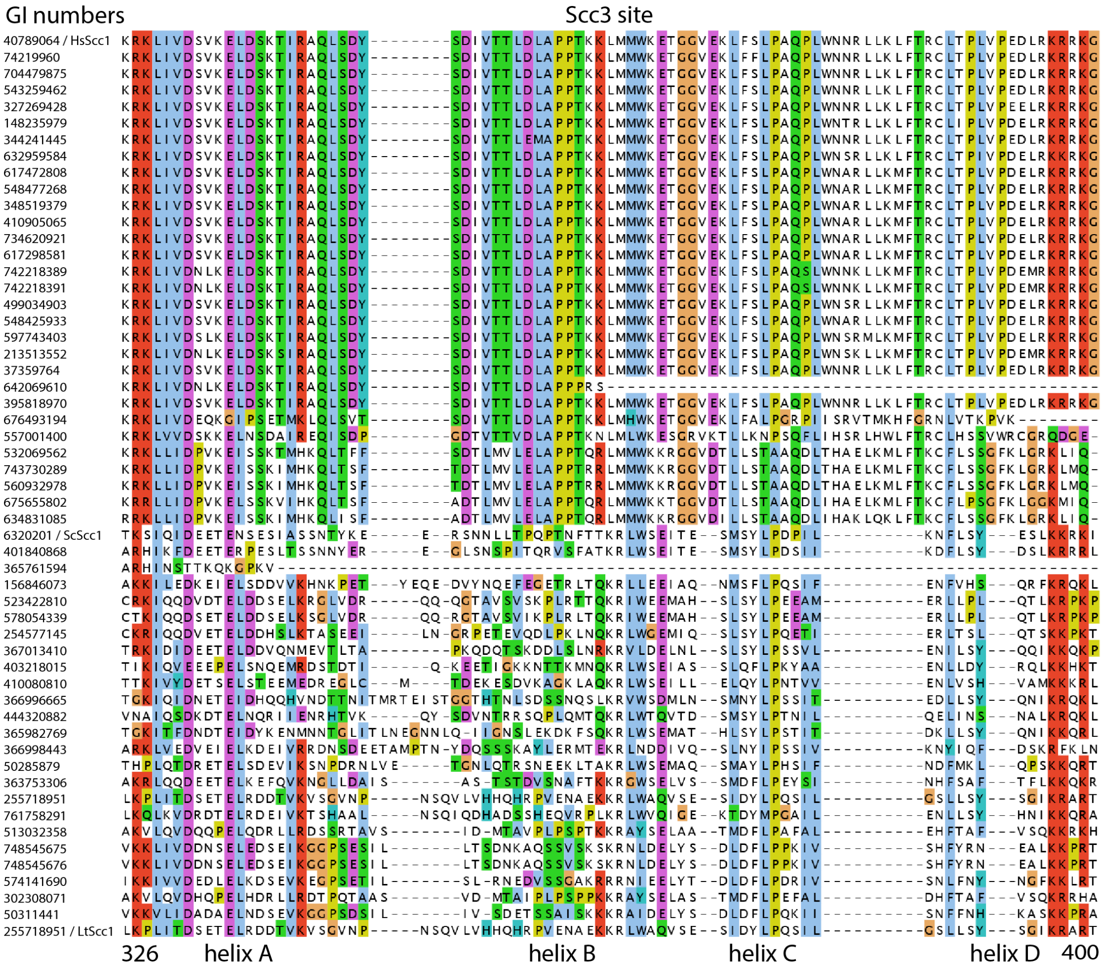

Supplementary Figure S4. ***S. cerevisiae*** **Pds5(Y458K) growth tests.** Related to Figures 2A & B. Wild type cells (K699, see list of strains) and cells with the endogenous *PDS5* locus deleted and expressing *PDS5* WT (K25118) or *pds5* (Y458K) (K25126) integrated at the *lys2* locus were streaked on YEPD plates and incubated at different temperatures. Note that ScPds5 Y458 corresponds to LtPds5 Y493 (labelled in Figure 1D).

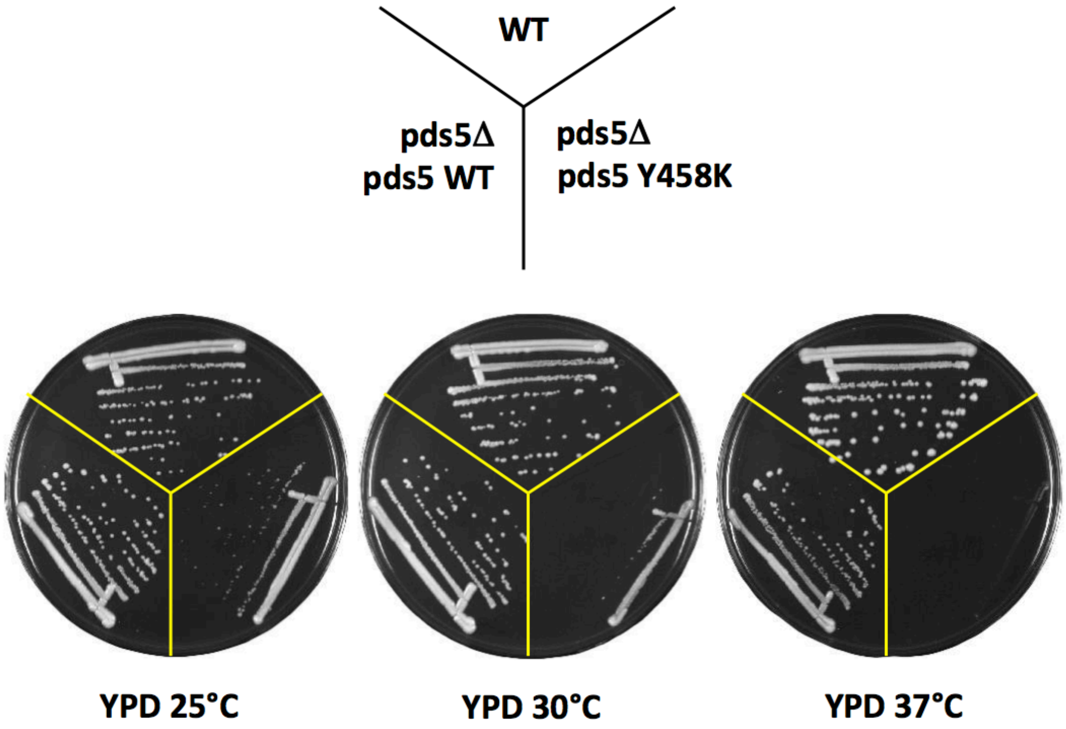

Supplementary Figure S5. Reduced occupancy of pds5(Y458K) across the genome. Related to Figure 2C. (A) Calibrated ChIP-seq profiles of Pds5 (strain K25120) and pds5(Y458K) (K25128) showing the number of reads at each base pair on chromosome I. (B) Calibrated ChIP-seq profiles showing the percentage of reads of pds5(Y458K) (K25128) at each base pair away from the CDEIII element, averaged over all sixteen chromosomes with respect to Pds5 (K25120). (C) FACS data showing cycling of the cells used in this figure and Figure 2C (strains K699, K25120, K25128).

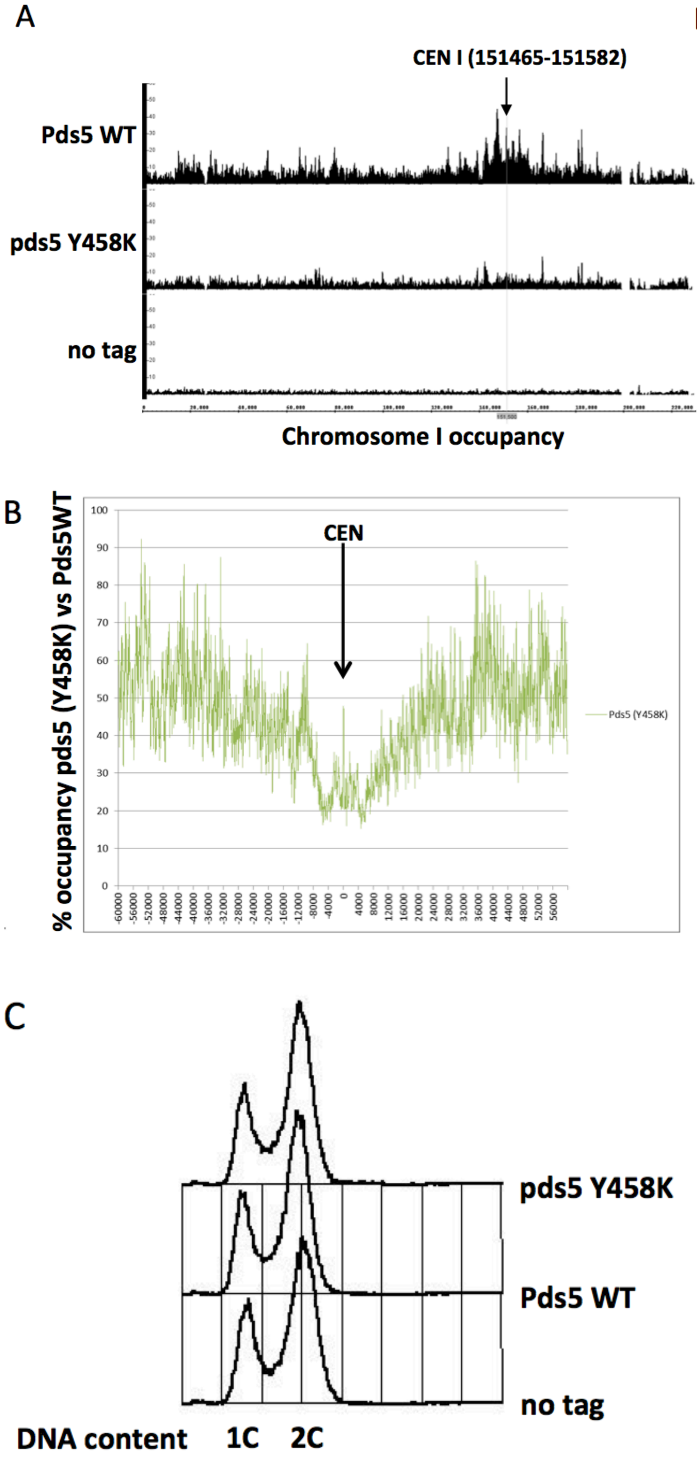

Supplementary Figure S6. Superposition of LtPds5 domains on previously determined Scc3 and SA2 structures. Related to Figure 3C. The LtPds5 structure was divided into four domains (left) that were independently aligned in 3D (PyMOL 1.7.6.2 'cealign' and 'align' commands) against the entire Scc3 (PDB 4UVK, middle) and SA2 (PDB 4PJU, right) structures. No manual adjustments were made. Two parts of Pds5 align reasonably well against their corresponding parts in both Scc3 and SA2. The C-terminal domain aligns less well and does not superimpose at all close to the C-terminus. It should be noted that the aligning parts show the highest conservation of the canonical HEAT repeat fold.

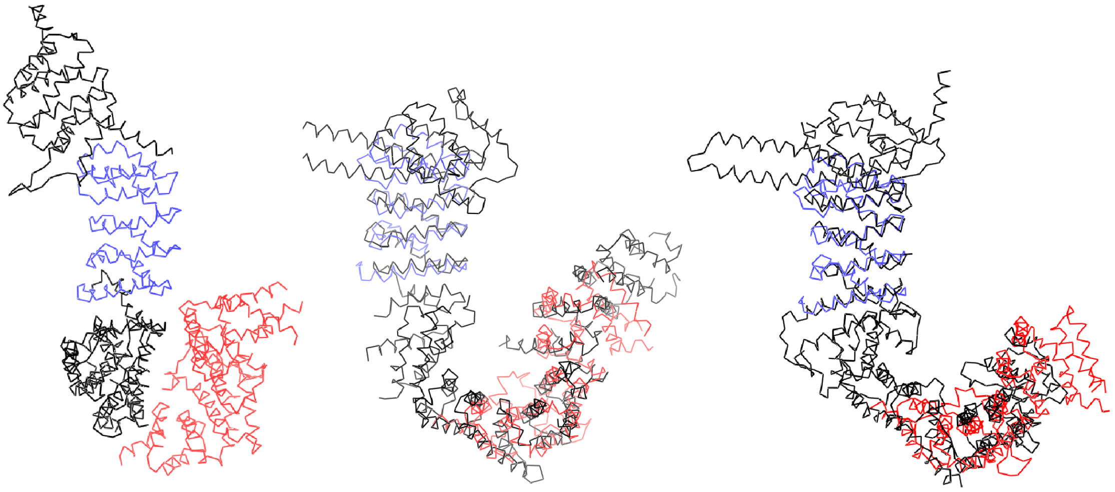

**Pds5**	**SA2**	**Scc3**
PDB 5F0O	PDB 4PJU	PDB 4UVK
N-terminal: 76-278	-	-
middle 1: 288-470	RMSD 3.3 Å	RMSD 3.4 Å
middle 2: 471-726	-	-
C-terminal: 763-1109	RMSD 5.1 Å	RMSD 5.5 Å

Supplemental Experimental Procedures

Cloning, overexpression and purification

Lachancea thermotolerans CBS6340 Pds5 (LtPds5, NCBI database identifier XP_002553028.1) was expressed in *E. coli* using a codon-optimised synthetic gene (Epoch Lifescience, TX). Two constructs were used, LtPds5-1, amino acids 35-1221, approx.. 137 kDa and LtPds5-2, amino acids 45-1221, approx. 135 kDa (see Table 1). These were cloned into expression vector pHis17 using Gibson assembly (New England Biolabs, MA), adding the affinity purification tag LHHHHHH at the C-termini. For overexpression, C41(DE3) cells (Lucigen, WI) were transformed with the resulting vectors and grown in 2xTY media and induced with 1 mM IPTG at 16˚C overnight. Cells were harvested and re-suspended in lysis buffer (50 mM Tris-HCl, 150 mM NaCl, 2 mM TCEP and 5% (w/v) glycerol, pH 8.0), and lysed through a Constant Systems cell disruptor at 25 kPSI in the presence of DNase, RNase and EDTA-free protease inhibitors (Roche). The cell lysates were clarified by ultracentrifugation at 200,000 g in a Beckman 45 Ti rotor and applied to nickel resin (5 ml HisTrap HP, GE Healthcare) and eluted with 50-150 mM imidazole in lysis buffer. Fractions containing Pds5 proteins were further purified using anion exchange chromatography (5 ml HiTrap Q FF, GE Healthcare) with gradients of 100-1000 mM NaCl in buffer containing 50 mM Tris-HCl, 100 mM NaCl, 2 mM TCEP, 5 % (w/v) glycerol, pH 8.5. Proteins were concentrated using spin concentrators (Vivaspin, Satorius, 50 kDa MWCO) and further purified using size exclusion chromatography (Sephacryl S300 16/60, GE Healthcare) in buffer containing 50 mM Tris-HCl, 250 mM NaCl, 5 mM TCEP, pH 7.5. Selenomethionine-labeled LtPds5 proteins were expressed using a published feedback inhibition procedure (van den Ent et al., 1999; Van Duyne et al., 1993) and purified using the same protocol for the native proteins. All purifications were performed at 4˚C. The LtScc1 (NCBI database identifier XP_002555756.1) peptide (residues 121-143: LTNPSQYLLQDAVTEREVLLVPG) design was informed by published results (Chan et al., 2013). Two otherwise identical, selenomethionine-substituted mutant peptides, Y127SeMet and L128SeMet, were used to confirm the

orientation of Scc1 polypeptide. All peptides were chemically synthesised (Generon, UK, Cambridge Peptides, UK, and Genscript, USA/Hong Kong).

Crystallisation and data collection

Initial crystallisation experiments were carried out using sitting-drop vapour diffusion with LMB's in-house high-throughput crystallisation facility at 100 nl volumes (Stock et al., 2005). LtPds5-2 protein (amino acids 45-1221) was crystallised with reservoir solutions containing 100 mM HEPES pH 7.5, and 1.3-1.6 M lithium sulphate, and both native and selenomethionine-substituted crystals were obtained in similar conditions. Diffraction quality crystals were grown at 20 °C using concentrations of 10 mg/ml, mixed with reservoir solution 0.3 - 0.4 times the protein solution's volume at 1.2 µl volumes. Crystals were observed after 2-3 weeks and were cryo-protected by serially increasing lithium sulphate concentration up to 2.2 M in the drop and subsequent flash freezing in liquid nitrogen. For Pds5-Scc1 co-crystallization, LtPds5-1 protein (amino acids 35-1221) at 8 mg/ml was mixed with LtScc1 peptide (amino acids 121-143) in five times molar excess. Complex crystals were obtained with reservoir solution containing 50 mM sodium cacodylate pH 6.5, 1.4 – 1.6 M ammonium sulphate and 5 mM magnesium acetate, and grew within a week. The crystals were cryo-protected by transferring to reservoir solution supplemented with 1.2 M sodium malonate and flash frozen in liquid nitrogen before data collection. Co-crystals of Pds5 and two selenomethionine-substituted Scc1 mutant peptides (Y127SeMet and L128SeMet) were grown with LtPds5-2 protein under reservoir solution containing 50 mM sodium cacodylate pH 6.5, 1.4 M lithium sulphate, 40 mM sodium citrate. The complex crystals were cryo-protected by serially increasing lithium sulphate in the drop and flash frozen in liquid nitrogen. Diffraction data were collected at 100 K on beamlines i03 at Diamond Light Source (Harwell, UK) and id23eh1 at the ESRF (Grenoble, France).

Structure determination

Diffraction data were integrated and scaled with XDS (Kabsch, 2010) and SCALA (Winn et al., 2011). Phasing was done by SeMet SAD combining data from two separate crystals in order to increase multiplicity and anomalous signal.

Selenium positions were identified and SAD phases were calculated using SHELXC/D/E (Sheldrick, 2008) and PHASER (McCoy et al., 2007). An initial atomic model was obtained using Crank2 (Skubak and Pannu, 2013), and manually improved using COOT (Emsley et al., 2010) and MAIN (Turk, 2013). For refinement, the high resolution native apo dataset (Table 1) was corrected for anisotropy using the UCLA Diffraction Anisotropy Server (services.mbi.ucla.edu/anisoscale/) (Strong et al., 2006), and the model was further rebuilt and refined in cycles at 3.2 Å resolution, manually rebuilt as above and refined with REFMAC (Murshudov et al., 1997) and PHENIX (Adams et al., 2010). The *Lt*Pds5-*Lt*Scc1 complex dataset was even more anisotropic and was also corrected using the UCLA server. Data extended to 3.5 Å in two directions (Table 1) and 4.5 in the third, leading to an estimate of overall resolution of 3.6 Å. Note that dataset statistics listed in Table 1 are those of the uncorrected data at 3.6 Å resolution before applying anisotropy correction. The complex structure was solved by molecular replacement with PHASER. Due to overall conformational changes between apo-Pds5 and the Pds5-Scc1 complex structures, molecular replacement was performed with the apo structure cut into 4 roughly equal sized subdomains. After improving the atomic model of Pds5 within the complex structure by manual building and refinement as described above, strong extra electron density was located and the LtScc1 peptide was fitted. Modelling of the Scc1 sequence at this low resolution was guided by two additional selenium SAD experiments (details of data not shown) using peptides containing SeMet residues in two positions: LtScc1(Y127SeMet) and LtScc1(L128SeMet). Phases were calculated using ANODE (Thorn and Sheldrick, 2011) and the refined Pds5 structure in the complex crystals. The entire model was then built and refined in cycles as for the apo structure. For R-factors and other statistics of the data and models, please refer to Table 1. Because of low resolution, no waters or ions were added to any of the structures. Figures were prepared using PYMOL (Schroedinger). Coordinates and structure factors were deposited in the Protein Data Bank (PDB) with accession numbers 5F0N (apo-LtPds5) and 5F0O (LtPds5:LtScc1 peptide complex).

Cell viability analysis of S. cerevisiae Pds5 and Scc1 mutants

The corresponding residues in *S. cerevisiae* Pds5 and Scc1 were located by sequence alignments. Mutant versions of Pds5 (under its native promoter) were incorporated at the *lys2* locus in heterozygous *PDS5/pds5Δ* diploid cells (K25105, see List of Strains in Supplementary Data). Diploids were sporulated on SpoVB media plates and tetrads dissected at 25°C on YPD media plates. The genotype of the resulting haploids was determined by replica plating, and viable cells with only the ectopic copy were additionally tested for temperature sensitivity streaking cells on YEP glucose plates at 25°C, 30°C and 37°C (Figure S4). Mutant versions of *scc1* (under its native promoter) were incorporated at the *leu2* locus in heterozygous *SCC1/scc1D* diploid cells (K12714) and analysed in a similar manner. All mutations were confirmed by DNA sequencing.

Co-immunoprecipitations

Strains were grown in YEPD at 25°C to OD_{600nm} = 0.7 and 70 OD units were washed in ice-cold PBS and frozen at -80°C. Pellets were thawed and re-suspended in lysis buffer (50 mM Tris/HCl, 100 mM NaCl, 5 mM EDTA, 1 mM DTT, 1 mM PMSF, Roche Complete Protease Inhibitors) and lysed in a FastPrep-24 (MP Biomedicals) disruptor 3 times for 1 min at 5.5 m/s with an equal volume of acid-washed glass beads (Sigma). Lysates were cleared by centrifugation at 13 Krpm for 30 min at 4°C and the protein amount of the supernatant was quantified with a Bradford assay. For cohesion immuneprecipitation, 150 µl of washed anti-HA High Affinity Matrix (Roche) was added to the cleared lysates and incubated over night at 4°C while rotating. The incubated anti-HA High Affinity Matrix was washed 3 times with 1 ml of lysis buffer, re-suspended in 60 µl of SDS-PAGE sample buffer and incubated at 95 °C for 10 min. 10 µl of each sample was loaded onto a precast Tris-acetate gel (3-8%, NuPAGE), followed by Western blotting and immunodetection of the PK epitopes with anti-PK antibody (AbD Serotec) and the HA epitopes with anti HA antibody (12CA5).

Calibrated ChIP-seq analysis

Calibrated ChIP-seq was performed as described (Hu et al., 2015) using K23308, K699, K25120 and K25128 strains for this assay.

Pds5 sequence conservation mapping on Pds5 crystal structure

500 sequences most similar to *L. thermotolerans* Pds5 were selected from a BLAST search and aligned using Clustal Omega (http://clustal.org), before mapping sequence conservation at each residue position onto the structure with ConSurf (http://consurf.tau.ac.il) (Ashkenazy et al., 2010).

List of Strains:

All yeast strains are derivatives of W303, except K23308.

K699 *MATa, ade2-1, trp1-1, can1-100, leu2-3,112,his3-11,15, ura3, GAL, psi+*

K12714 *MATa/alpha scc1:KanMx / WT*

K23308 *C.glabrata, MATa, Scc1PK9::NatMX*

K24593 *MATalpha, scc1:KanMX, Scc1-HA6 in pRS305H*

K24595 *MATalpha, leu2:Scc1-HA6:leu2*

K24958 *MATa/alpha, scc1:KanMX / WT, leu2:Scc1(V137K)-HA6:leu2*

K25002 *MATa, leu2:Scc1(V137K)-HA6:leu2*

K25105 *MATa/alpha pds5::HIS / WT*

K25106 *MATa/alpha, pds5::HIS / WT, lys2:Pds5-PK9-HphMX:lys2*

K25108 *MATa/alpha, pds5::HIS / WT, lys2:Pds5(Y458K)-PK9-HphMX:lys2*

K25118 *MATa pds5::HIS, lys2:Pds5-PK9-HphMX:lys2*

K25120 *MATa, lys2:Pds5-PK9-HphMX:lys2*

K25126 *MATa pds5::HIS, lys2:Pds5(Y458K)-PK9-HphMX:lys2*

K25128 *MATa, lys2:Pds5(Y458K)-PK9-HphMX:lys2*

K25166 *MATa/alpha scc1:KanMX / WT, leu2:Scc1-HA6:leu2*

K25202 *MATa, pds5::HIS, lys2:Pds5-PK9-HphMX:lys2, leu2:Scc1-HA6:leu2*

K25204 *MATalpha, lys2:Pds5-PK9-HphMX:lys2, scc1:KanMX, leu2:Scc1-HA6:leu2*

K25206 *MATalpha, pds5::HIS, lys2:Pds5-PK9-HphMX:lys2, leu2:Scc1(V137K)-HA6:leu2*

K25210 *MATalpha, lys2:Pds5(Y458K)-PK9-HphMX:lys2, scc1:KanMX, leu2:Scc1-HA6:leu2*

Supplemental References

Adams, P. D., Afonine, P. V., Bunkoczi, G., Chen, V. B., Davis, I. W., Echols, N., Headd, J. J., Hung, L. W., Kapral, G. J., Grosse-Kunstleve, R. W., McCoy, A. J., Moriarty, N. W., Oeffner, R., Read, R. J., Richardson, D. C., Richardson, J. S., Terwilliger, T. C., and Zwart, P. H. (2010). PHENIX: a comprehensive Python-based system for macromolecular structure solution. Acta Crystallogr D Biol Crystallogr *66*, 213-221.

Ashkenazy, H., Erez, E., Martz, E., Pupko, T., and Ben-Tal, N. (2010). ConSurf 2010: calculating evolutionary conservation in sequence and structure of proteins and nucleic acids. Nucleic Acids Res. *38*, W529-W533.

Chan, K. L., Gligoris, T., Upcher, W., Kato, Y., Shirahige, K., Nasmyth, K., and Beckouet, F. (2013). Pds5 promotes and protects cohesin acetylation. Proc. Natl. Acad. Sci. U. S. A. *110*, 13020-13025.

Emsley, P., Lohkamp, B., Scott, W. G., and Cowtan, K. (2010). Features and development of Coot. Acta Crystallogr D Biol Crystallogr *66*, 486-501.

Hu, B., Petela, N., Kurze, A., Chan, K. L., Chapard, C., and Nasmyth, K. (2015). Biological chromodynamics: a general method for measuring protein occupancy across the genome by calibrating ChIP-seq. Nucleic Acids Res. *43*, e132.

Kabsch, W. (2010). XDS. Acta Crystallogr D Biol Crystallogr *66*, 125-132.

McCoy, A. J., Grosse-Kunstleve, R. W., Adams, P. D., Winn, M. D., Storoni, L. C., and Read, R. J. (2007). Phaser crystallographic software. J. Appl. Crystallogr. *40*, 658-674.

Murshudov, G. N., Vagin, A. A., and Dodson, E. J. (1997). Refinement of macromolecular structures by the maximum-likelihood method. Acta Crystallogr D Biol Crystallogr *53*, 240-255.

Sheldrick, G. M. (2008). A short history of SHELX. Acta Crystallogr A *64*, 112-122.

Skubak, P., and Pannu, N. S. (2013). Automatic protein structure solution from weak X-ray data. Nat Commun *4*, 2777.

Stock, D., Perisic, O., and Löwe, J. (2005). Robotic nanolitre protein crystallisation at the MRC Laboratory of Molecular Biology. Prog Biophys Mol Biol *88*, 311-327.

Strong, M., Sawaya, M. R., Wang, S., Phillips, M., Cascio, D., and Eisenberg, D. (2006). Toward the structural genomics of complexes: crystal structure of a PE/PPE protein complex from Mycobacterium tuberculosis. Proc. Natl. Acad. Sci. U. S. A. *103*, 8060-8065.

Thorn, A., and Sheldrick, G. M. (2011). ANODE: anomalous and heavy-atom density calculation. J. Appl. Crystallogr. *44*, 1285-1287.

Turk, D. (2013). MAIN software for density averaging, model building, structure refinement and validation. Acta Crystallogr D Biol Crystallogr *69*, 1342-1357.

van den Ent, F., Lockhart, A., Kendrick-Jones, J., and Löwe, J. (1999). Crystal structure of the N-terminal domain of MukB: a protein involved in chromosome partitioning. Structure *7*, 1181-1187.

Van Duyne, G. D., Standaert, R. F., Karplus, P. A., Schreiber, S. L., and Clardy, J. (1993). Atomic structures of the human immunophilin FKBP-12 complexes with FK506 and rapamycin. J. Mol. Biol. *229*, 105-124.

Winn, M. D., Ballard, C. C., Cowtan, K. D., Dodson, E. J., Emsley, P., Evans, P. R., Keegan, R. M., Krissinel, E. B., Leslie, A. G., McCoy, A., McNicholas, S. J., Murshudov, G. N., Pannu, N. S., Potterton, E. A., Powell, H. R., Read, R. J., Vagin, A., and Wilson, K. S. (2011). Overview of the CCP4 suite and current developments. Acta Crystallogr D Biol Crystallogr *67*, 235-242.
