# Supplementary material for: Crystal Structure of the Cohesin Gatekeeper Pds5 and in Complex with Kleisin Scc1
Source: Cell Rep. 2016 Feb 25;14(9):2108–15. doi: 10.1016/j.celrep.2016.02.020 (PMC4793087; doi:10.1016/j.celrep.2016.02.020)
Supplement: Document S2. Article plus Supplemental Information [file mmc2.pdf]

Cell Reports

Report

Crystal Structure of the Cohesin Gatekeeper Pds5 and in Complex with Kleisin Scc1

Graphical Abstract

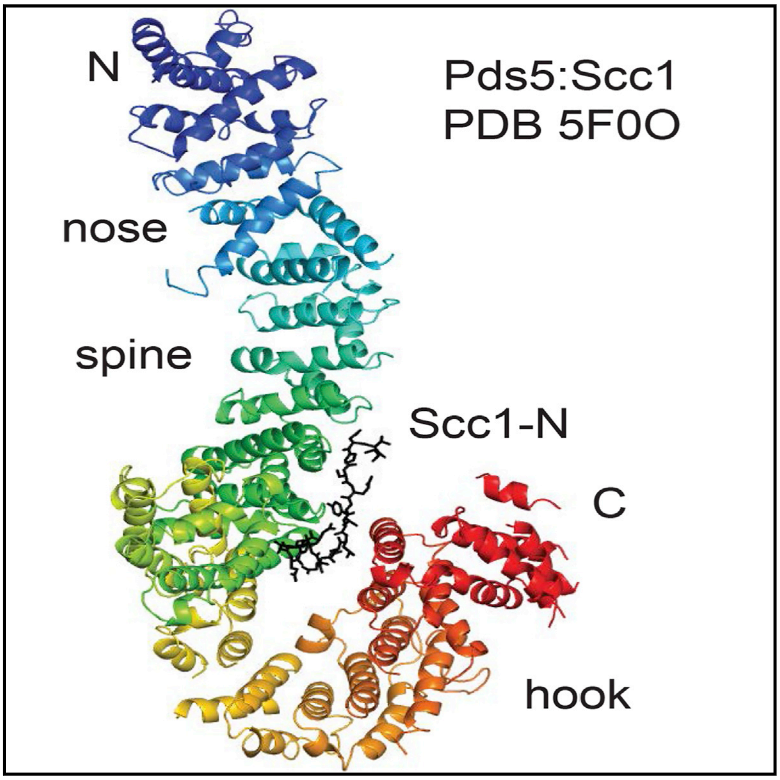

Authors

Byung-Gil Lee, Maurici B. Roig, Marijke Jansma, Naomi Petela, Jean Metson, Kim Nasmyth, Jan Löwe

Correspondence

jyl@mrc-lmb.cam.ac.uk

In Brief

Cohesin entraps DNA in a ring, facilitating sister chromatid cohesion during mitosis and meiosis. Lee et al. describe the structure of the cohesin subunit Pds5 in complex with Scc1, cohesin's kleisin, mutants disrupting the Pds5:Scc1 interaction, and structural similarity to another cohesin subunit, Scc3.

Highlights

- The crystal structure of the cohesin subunit Pds5 was determined
- The crystal structure of Pds5 in complex with Scc1 binding region was determined
- Structure-based mutants in Pds5 and Scc1 were analyzed by coIP and ChIP-seq
- Pds5 shows some similarity to Scc3, the other large HEAT repeat cohesin subunit

Accession Numbers

5F0N
5F0O

Lee et al., 2016, Cell Reports *14*, 2108–2115
March 8, 2016 ©2016 The Authors
http://dx.doi.org/10.1016/j.celrep.2016.02.020

CellPress

OPEN ACCESS
CellPress

Cell Reports
Report

Crystal Structure of the Cohesin Gatekeeper Pds5 and in Complex with Kleisin Scc1

Byung-Gil Lee,[1] Maurici B. Roig,[2] Marijke Jansma,[1] Naomi Petela,[2] Jean Metson,[2] Kim Nasmyth,[2] and Jan Löwe[1,*]
[1]MRC Laboratory of Molecular Biology, Cambridge CB2 0QH, UK
[2]Department of Biochemistry, University of Oxford, Oxford OX1 3QU, UK
*Correspondence: jyl@mrc-lmb.cam.ac.uk
http://dx.doi.org/10.1016/j.celrep.2016.02.020
This is an open access article under the CC BY license (http://creativecommons.org/licenses/by/4.0/).

SUMMARY

Sister chromatid cohesion is mediated by cohesin, whose Smc1, Smc3, and kleisin (Scc1) subunits form a ring structure that entraps sister DNAs. The ring is opened either by separase, which cleaves Scc1 during anaphase, or by a releasing activity involving Wapl, Scc3, and Pds5, which bind to Scc1 and open its interface with Smc3. We present crystal structures of Pds5 from the yeast *L. thermotolerans* in the presence and absence of the conserved Scc1 region that interacts with Pds5. Scc1 binds along the spine of the Pds5 HEAT repeat fold and is wedged between the spine and C-terminal hook of Pds5. We have isolated mutants that confirm the observed binding mode of Scc1 and verified their effect on cohesin by immunoprecipitation and calibrated ChIP-seq. The Pds5 structure also reveals architectural similarities to Scc3, the other large HEAT repeat protein of cohesin and, most likely, Scc2.

INTRODUCTION

The segregation of multiple chromosomes during mitosis in eukaryotes is made possible by sister chromatid cohesion whose destruction triggers the simultaneous disjunction of all sister chromatid pairs at the metaphase-to-anaphase transition. Cohesion is mediated by the cohesin complex, at whose core is a heterodimer of coiled-coil Smc1 and Smc3 proteins (structural maintenance of chromosomes), each with a hinge dimerization domain at one end and an ABC ATPase head domain at the other (Nasmyth and Haering, 2009; Onn et al., 2008). The latter are bridged by the alpha kleisin Scc1, forming a molecular ring up to 50 nm in contour length, within which sister DNA can be entrapped (Haering et al., 2002, 2008). Anaphase is triggered through cleavage of Scc1 by the protease separase, whose activity is regulated through the cell-cycle-specific destruction of an inhibitor securin by the anaphase-promoting complex/cyclosome (APC/C) (Uhlmann et al., 1999). Scc1 cleavage opens the cohesin ring, permitting traction of sister chromatids to opposite poles through pulling forces associated with microtubules attached to kinetochores. Loading of cohesin onto chromosomes, as well as separase-independent unloading (release), depends on several other proteins, Scc3, Pds5, Wapl, and Scc2/4, that interact with the core ring subunits (Ciosk et al., 2000; Hartman et al., 2000; Kueng et al., 2006; Losada et al., 2000; Panizza et al., 2000; Rankin et al., 2005; Schmitz et al., 2007; Tóth et al., 1999).

Cohesin loading requires ATP hydrolysis, Scc3, and Scc2/4 (Arumugam et al., 2003, 2006; Ciosk et al., 2000; Hu et al., 2011; Weitzer et al., 2003). Loading is believed to require opening of a gate created by transient dissociation of the Smc1/3 hinge interface (Gruber et al., 2006). Unloading occurs through two mechanisms: in addition to irreversible opening of the ring by separase, cohesin has a "releasing activity" that enables a dynamic association with chromosomes, in particular during G1 phase of the cell cycle (Gandhi et al., 2006; Kueng et al., 2006; Nishiyama et al., 2010). Though active throughout the cell cycle, releasing activity is most active, at least in animal cells, during mitosis, when it is responsible for removing a large fraction of cohesin from chromosome arms in the prophase pathway (Losada et al., 1998; Waizenegger et al., 2000). Centromeric cohesin is protected from the prophase pathway by shugoshin-mediated recruitment of PP2A to centromeres (Riedel et al., 2006; Tang et al., 2006).

The observation that releasing activity is abolished by fusing Scc1's N terminus to Smc3's C terminus suggests that release occurs through the escape of DNA from the ring through a gate between Scc1's N-terminal domain and Smc3's coiled coil (see Figure 2B for an overview) (Chan et al., 2012). For releasing activity not to destroy all sister chromatid cohesion, it is countered by acetylation during S phase of a pair of lysine residues in Smc3's ATPase head by the Eco1 acetyl transferase (Ivanov et al., 2002; Nishiyama et al., 2010; Rolef Ben-Shahar et al., 2008; Rowland et al., 2009; Unal et al., 2008).

Consistent with the notion that the main role of acetylation is to block release, mutants defective in releasing activity enable cells to proliferate in the absence of Eco1 (Rolef Ben-Shahar et al., 2008; Rowland et al., 2009; Sutani et al., 2009; Tanaka et al., 2001). Indeed, most releasing activity mutations, be they in Smc3, Pds5, Scc3, or Wapl, were initially identified as *eco1* suppressors. Smc3 deacetylation, which is mediated by Hos1 in yeast (Beckouët et al., 2010; Borges et al., 2010; Xiong et al., 2010) and HDAC8 in mammalian cells (Deardorff et al., 2012), takes place upon Scc1 cleavage at the onset of anaphase.

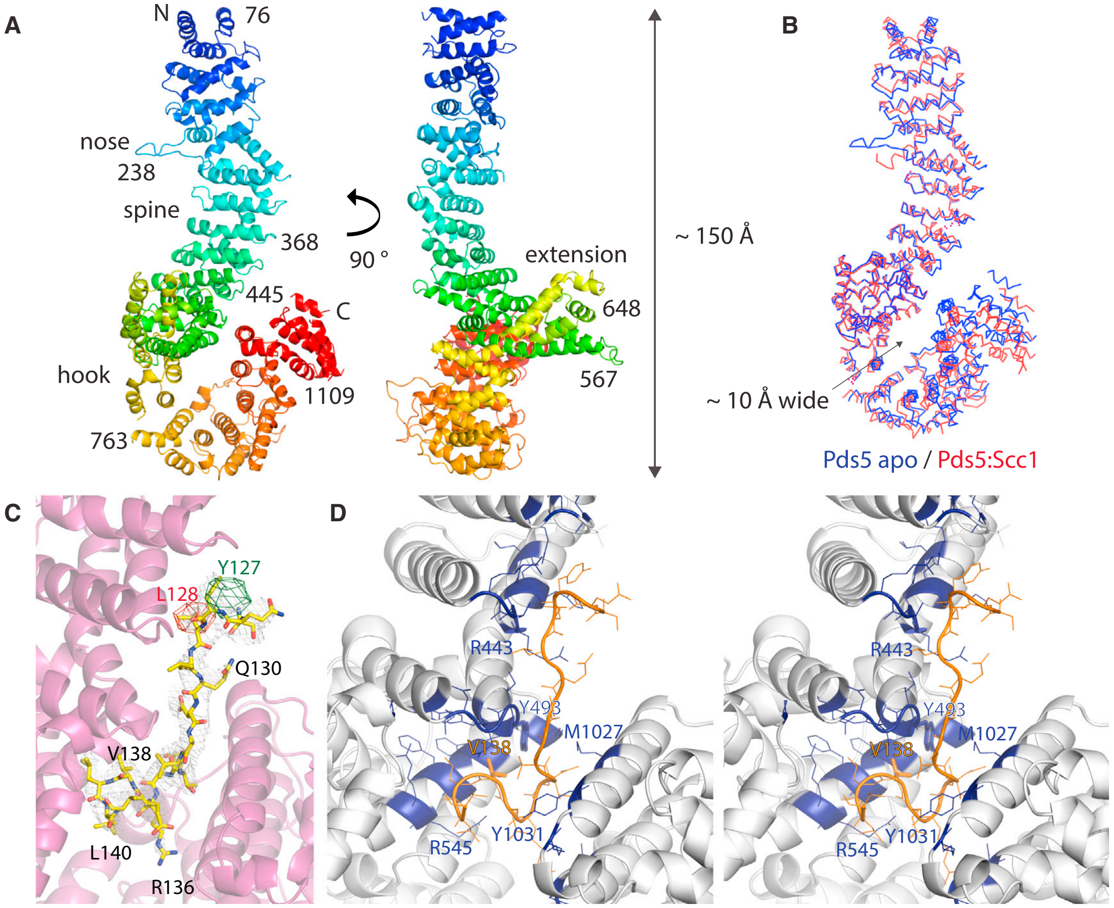

Figure 1. Crystal Structures of Pds5 from *L. thermotolerans* in the Apo Form and Bound to Scc1

(A) Crystal structure of Pds5 from *L. thermotolerans*. Residues are colored from the N terminus (blue) to the C terminus (red). Pds5 is composed of a large number of HEAT repeats, many of them irregular to produce protrusions, such as the nose and the extension domain, that consist of helices that are additions to the regular HEAT repeat. The N and C termini lie at opposite ends of the 150-Å-long molecule. The C-terminal hook bends back, creating a contact between the hook and the spine and forming a small ring.

(B) Conformational changes between Pds5 in the apo form and when bound to a peptide containing Scc1's Pds5-binding region. Because the Scc1 peptide is wedged between the hook and the spine, the hook slightly widens upon Scc1 binding.

(C) Close-up of the 2Fo-Fc electron density map and the fitted Scc1 peptide that extends from residue 125 to 141. The orientation here is similar to that in (A) and (B). The densities shown in green and red are the result of two separate SeMet SAD experiments with Scc1 peptides that contained SeMet residues at the indicated positions Scc1(Y127SeMet) and Scc1(L128SeMet), revealing the sequence and direction of the fitted peptide.

(D) Stereo view of the bound Scc1 peptide. Key binding residues in Pds5 are highlighted in blue. Most notably, M1027 and Y1031 on the hook (right) also mediate contact between the hook and spine (left) in Pds5 apo. On the spine, interacting residues include I403, R410, R443, E444, T445, R446, Y492, Y493, I494, N495, K535, S538, S539, A542, F543, and R545. We found two residues that compromised the viability of yeast strains when the corresponding residues were mutated: Scc1(V138) and Pds5(Y493) (Figure 2A).

See also Figures S1–S3.

In addition to being crucial for the release of cohesin from chromosomes, during S phase, Pds5 promotes the acetylation of Smc3 that will protect cohesin from releasing activity in G2 (Chan et al., 2013; Vaur et al., 2012). It also prevents deacetylation by Hos1, from S phase until Scc1 cleavage at the onset of anaphase. Lastly, at least in yeast, Pds5 has a role in maintaining sister chromatid cohesion during G2/M by a mechanism that does not involve Smc3 acetylation (Chan et al., 2013; Tong and Skibbens, 2014). Thus, Pds5 can be considered the gatekeeper of the cohesin ring. A complementary study investigating the interaction of Pds5 and Scc1 and the structure of their complex is reported in this issue of *Cell Reports* (Muir et al., 2016).

RESULTS AND DISCUSSION

Structure of *Lachancea thermotolerans* Pds5

We expressed Pds5 from *Lachancea thermotolerans* (Lt) in *E. coli* and determined its structure at 3.2 Å resolution (Experimental Procedures; Figures 1A and S1). LtPds5 is 47% identical in sequence to *Saccharomyces cerevisiae* (Sc) Pds5. The large molecule is exclusively alpha helical, composed of a large

Table 1. Crystallographic Data

Statistics			
Sample	*L. thermotolerans* Pds5 SeMet	*L. thermotolerans* Pds5 Native	*L. thermotolerans* Pds5:Scc1 Complex Native
NCBI Database IDs	XP_002553028.1	XP_002553028.1	XP_002553028.1, XP_002555756.1
Constructs	M-45-1221-LHHHHHH	M-45-1221-LHHHHHH	M-35-1221-LHHHHHH, Scc1 peptide 121-143: LTNPSQYLLQDAV TEREVLLVPG
Data Collection			
Beamline	Diamond I03	ESRF id23eh1	ESRF id23eh1
Wavelength (Å)	0.97941	0.97960	0.97949
Method	SeMet SAD	isomorphous to SeMet	molecular replacement
Crystal			
Space group	H3	H3	H3
Cell (Å)	237.5, 237.5, 80.5, 120°	238.2, 238.2, 80.7, 120°	235.7, 235.7, 94.4, 120°
Scaling			
Resolution (Å)	3.2	3.2	3.6
UCLA anisotropy (Å)[a]	na	3.2, 3.2, 3.5	3.5, 3.5, 4.5
Completeness (%)[b]	100.0 (100.0)	100.0 (100.0)	99.9 (99.9)
Multiplicity[b]	16.4 (16.5) merged two crystals	10.5 (10.8) one crystal	5.2 (5.4) one crystal
(I)/σ(I)[b]	15.5 (2.5)	14.5 (2.3)	11.2 (1.4)
R_{merge}[b]	0.133 (1.385)	0.109 (1.141)	0.079 (1.188)
R_{pim}[b]	0.050 (0.513)	0.056 (0.365)	0.060 (0.891)
CC1/2[b]	0.999 (0.884)	0.999 (0.871)	0.999 (0.682)
Anomalous correlation[b]	0.719 (0.048)		
Selenium sites	15 (100%)		
Refinement			
R/R_{free}[c]		0.236/0.295	0.232/0.291
Model		76–278, 288–688, 692–726, 736–751, 763–1067, 1072–1109, 40 unsequenced residues at N and C termini, no waters	80–278, 287–688, 692–726, 736–751, 763–1067, 1072–1109, 40 e at N and C, Scc1 peptide 125–141 no waters
Bond length RMSD (Å)		0.003	0.005
Bond angle RMSD (°)		0.726	0.837
Favored (%)[d]		99.5	98.4
Disallowed (%)[d]		0.2	0.3
MOLPROBITY score		100th percentile	99th percentile
PDB IDs		5F0N	5F0O

[a]Correction for anisotropy applied through online server (http://services.mbi.ucla.edu/anisoscale/). Resolution limits along the a*, b* and c* directions are listed.
[b]Values in parentheses refer to the highest recorded resolution shell.
[c]5% of reflections were randomly selected before refinement and kept throughout all procedures.
[d]Percentage of residues in regions of the Ramachandran plot (PROCHECK).

number of HEAT-like repeats and helical extensions/additions that deviate from the HEAT repeat pattern. The HEAT repeat pattern leads to a linear path from the N terminus to the C terminus, separating them by more than 100 Å. Deviations from the HEAT repeat pattern create a nose and extension domain (Figure 1A), as well as a very pronounced hook, bending back so that the most C-terminal portion of Pds5 contacts the middle section, which contains the most regular HEAT repeats and which we called the spine. Bending back the hook creates a small loop or ring with an inner diameter of approximately 10 Å.

Structure of Pds5 Bound to Scc1

The region within kleisin Scc1 of cohesin that binds Pds5 has been mapped previously (Chan et al., 2013), and we therefore synthesized a 23-amino-acid-long peptide from *L. thermotolerans* Scc1 (121–143) containing this region. Although binding was weak (Chan et al., 2013), molecular replacement and a difference Fourier map with data collected from co-crystals of LtPds5 containing the LtScc1 peptide revealed clear difference density. We could locate and model residues 125–141 of Scc1 in the density (Figure 1C; Table 1), using data from two SeMet

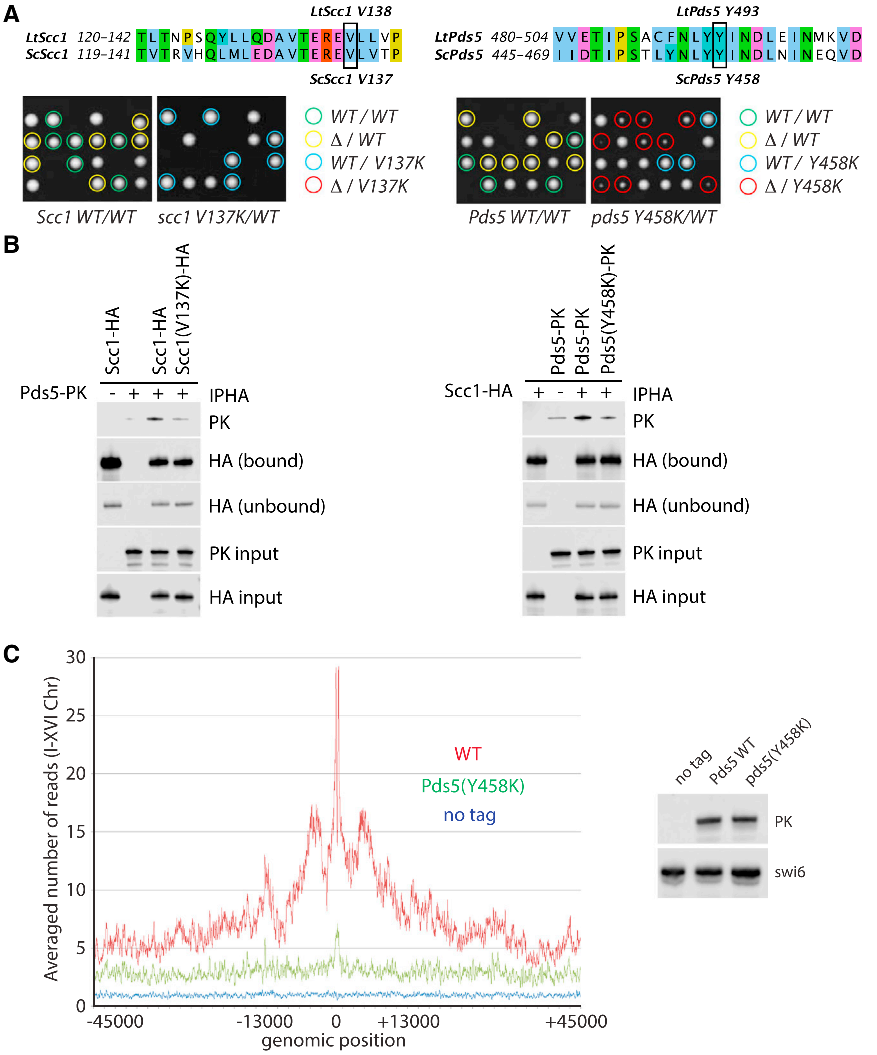

Figure 2. Analysis and Validation of the Pds5:Scc1 Interaction in *S. cerevisiae*

(A) Validation of the Pds5:Scc1 complex through yeast mutant viability analysis in *S. cerevisiae*: tetrad dissection of *Sc*Scc1(V137K) and *Sc*Pds5(Y458K). Sequence alignments indicate equivalent residues in *L. thermotolerans* and *S. cerevisiae*. Left: heterozygous diploids with one endogenous *SCC1* locus deleted (strain K12714) carrying either wild-type (K25166) or mutant *scc1 V137K* (K24958) genes integrated at the *leu2* locus were sporulated on YPD plates and four-spored asci dissected. Right: heterozygous diploids with one endogenous *PDS5* locus deleted (K25105) carrying either wild-type (K25106) or mutant *pds5 Y458K* (K25108) genes integrated at the *lys2* locus were sporulated on YPD plates and four-spored asci dissected. The resultant genotypes are color-coded. Note that strains expressing just scc1(V137K) or pds5(Y458K) (K25126) are lethal or sick, respectively, but neither of these mutations cause a dominant-negative effect when co-expressed with Scc1 WT (K25002) or Pds5 WT (K25120).

(B) Immunoprecipitation of Scc1 and detection of the co-precipitated Pds5 showing that scc1(V137K) (K24595, K25118, K25202, and K25206) and pds5(Y458K) (strains K24593, K25120, K25204, and K25210) greatly reduce the interaction with Pds5 and Scc1, respectively.

(C) Calibrated ChIP-seq profiles of Pds5 (strain K25120) and pds5(Y458K) (K25128) showing the number of reads at each base pair away from the CDEIII element averaged over all 16 chromosomes. Right: demonstration of equal Pds5 protein levels in those strains by western blotting. A non-averaged profile, a difference plot, and fluorescence-activated cell sorting data showing cycling cells are shown in Figures S5A–S5C.

See also Figures S4 and S5.

single-wavelength anomalous diffraction (SAD) datasets from crystals with Scc1 peptides containing SeMet residues to confirm the sequence assignment (Figure 1C, red and green densities).

Molecular replacement with four fragments was required because Pds5 shows changes in conformation upon peptide binding (Figure 1B). The hook opens and the entire structure bends such that the most N-terminal part (Figure 1B, left, top) moves by up to 10 Å relative to the central part (superposition based on Cα atoms of residues 473 to 726). The nose becomes disordered in the co-crystals.

In the Pds5:Scc1 complex structure, the Pds5 hook opens because the Scc1 peptide is wedged between the spine and the end of the hook, maintaining a closed-ring architecture (Figure 3C, bottom). Acidic and hydrophobic residues that in the apo structure of Pds5 make the contact with the spine, D999, M1027 and Y1031, now interact with the Scc1 peptide (Figure 1D). On the Pds5 spine many Pds5 amino acid side chains are in contact with the Scc1 peptide, as shown and listed in Figure 1D. Substitution by lysine of the residue equivalent to LtY493 in *S. cerevisiae*, namely, Y458K, greatly reduced proliferation and caused temperature-sensitive lethality (Figures 2A, right, and S4). In contrast, mutating the hydrophobic residues M1027 and Y1031 (located on the hook, I998 and F1002 in Sc) to lysine did not lead to lethality.

In the complex, Scc1 is in a mostly extended conformation, except toward its C terminus, which forms a more compact arrangement with helical turns. We found that V138 mutated to lysine was lethal in *S. cerevisiae* ScScc1(V137K) (Figure 2A, left), confirming previous results (Chan et al., 2013). In contrast, other mutations, namely, ScScc1 L126K, L128E, V132K, T133K, E134K, and E136K, had little or no effect (corresponding to Lt L128K, L129E, V133K, T134K, E135K, and E137K). As is indicated in Figure 1D, Scc1 V138 sits in a deep pocket in Pds5, lined by Pds5 Y493 and other hydrophobic residues.

To verify that the mutations function through a specific effect on the cohesin complex in cells, we performed immunoprecipitations of labeled cohesin subunits, expressed from endogenous promoters with and without the mutations ScScc1(V137K) and ScPds5(Y458K), determining the amounts of co-precipitated Pds5 and Scc1 by western blotting (Figure 2B). In both

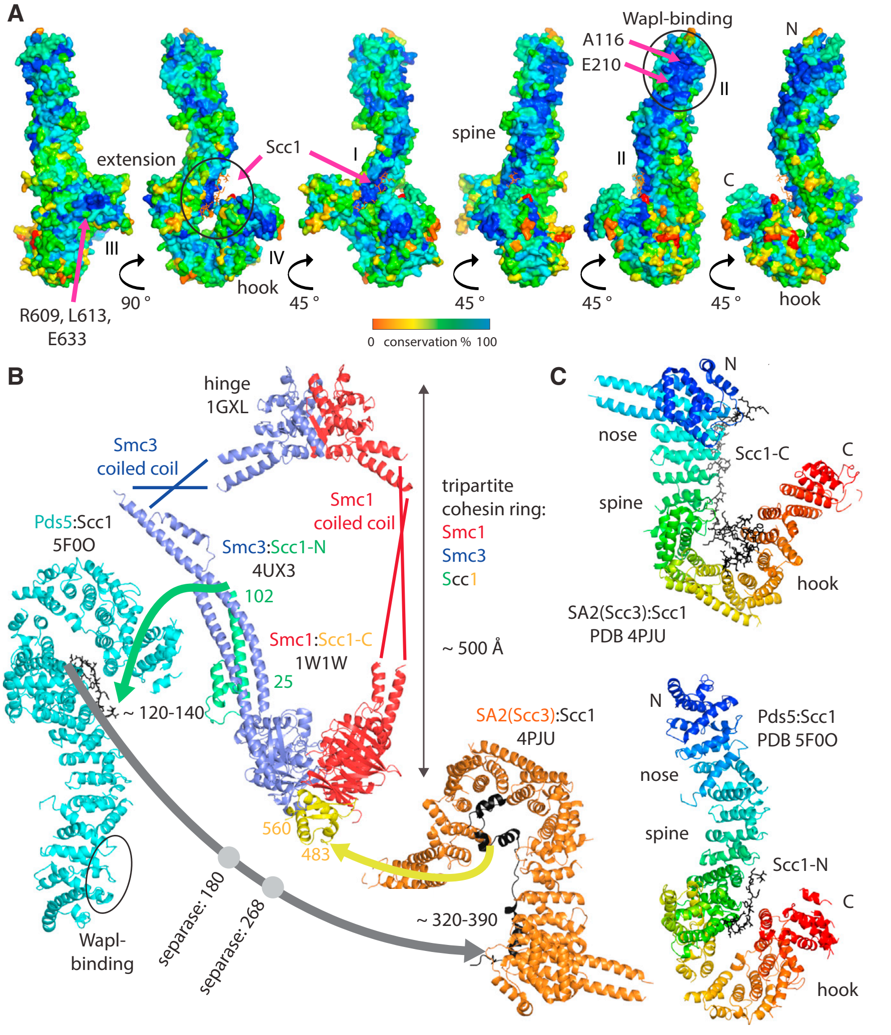

Figure 3. The Pds5 Structure in the Context of the Cohesin Complex

(A) Sequence conservation mapped onto the molecular surface of Pds5 in the Scc1-bound form. The Scc1 peptide is highlighted with arrows and is shown in orange. Four major areas of strong conservation have been detected: the Scc1 binding site on the spine and hook, validating Scc1's binding site on Pds5 (I). Extending toward the N terminus upward, conservation runs all the way from region I along the spine to the N-terminal domain, constituting region II. Previous mutants in Pds5 that were shown to reduce Wapl recruitment (Rowland et al., 2009) indicate that Wapl likely binds to the N-terminal region of Pds5 as indicated. Two additional patches are on the back of the extension domain (III) and at the tip of the hook (IV). Relative rotations are indicated by arrows, the panel on the left corresponds to the right panel in Figure 1A.

(B) To-scale exploded view drawing of the cohesion complex, including structurally known parts. The basic tripartite ring is made out of Smc1 (red) (Haering et al., 2004) and Smc3 (blue) (Gligoris et al., 2014) that dimerize through their hinge domains (top) (Haering et al., 2002). The Smc coiled-coil regions without crystal structures are indicated by lines and are not to scale. Kleisin Scc1 bridges the Smc ATPase head domains that are forming a heterodimer primed for ATP hydrolysis. The Smc3 head binds Scc1's N-terminal domain (green). Smc1 head domain binds the C-terminal domain of Scc1 (yellow). Residues in the middle of Scc1 are indicated by arrows as they have not been resolved by crystal structures, including the protease sites for separase. N-terminal to those separase sites, very close to the Scc1 site that binds the Smc3 head, lies the Scc1 site that binds between the hook and spine of Pds5. C-terminal to the separase sites is the site that binds between the hook and spine of Scc3 (Hara et al., 2014; Roig et al., 2014). Given that both Pds5 and Scc3 exhibit strong sequence conservation outside the known Scc1 binding surfaces, it is likely that these represent binding sites for Wapl and possibly sites on the Smc proteins. The Scc2/4 loading complex also has to interact with cohesin as loading proceeds through the hinge domains. Separase-independent unloading (releasing activity) most likely occurs through opening of the Smc3:Scc1-N interface, and it is therefore not surprising given the shown relative approximate positions that Pds5 has been implicated in the releasing activity. Note that the exact orientations of Pds5 and Scc1 with respect to the tripartite ring are not known and the drawing is not the result of docking calculations.

(C) Scc3/SA and Pds5 share overall architecture, including Scc1 binding. Both proteins are part of the cohesin complex and bind to cohesin's kleisin. Scc3 and Pds5 are large, irregular HEAT repeat proteins that separate N and C termini by large distances. Scc1 binding occurs mostly between the hook and spine in both proteins, creating a smaller ring in Scc3 than in Pds5. The extension and nose are less well conserved but still discernable. The nose is disordered in the Scc1-bound crystal form of Pds5 but visible in the apo form (Figure 1A, left). See also Figure S6 for subdomains of Pds5 aligned against Scc3/SA2.
See also Figure S6.

cases, a marked reduction was detected, more so with the Scc1 mutation. Calibrated chromatin immunoprecipitation sequencing (ChIP-seq) (Hu et al., 2015) showed that the ScPds5(Y458K) mutant greatly reduced Pds5's association with chromosomal cohesin, especially in pericentric sequences (Figure 2C). We showed previously that this is not due to a defect in cohesin loading onto chromosomes since Scc1(V137K), defective in Pds5 recruitment, does not affect loading (Chan et al., 2013).

We conclude that both Scc1(V138) and Pds5(Y493) are required for the Pds5:Scc1 interaction and that our structure reflects this interaction well.

Other Pds5 Interaction Regions

When we plotted sequence conservation among Pds5 homologs onto the Pds5 structure, several regions of potential functional interest became apparent (Figure 3A). The Scc1 peptide binding surfaces on Pds5, both on the hook and on the spine are well conserved, as expected for a binding site (region I). Extending along one edge of the spine, region II runs all the way to the N-terminal region at the top, where it ends with a large patch of conservation. This region includes a loop with consensus sequence APDAP (residues 116–120 in LtPds5). Regions III and IV are located on the extension domain and hook respectively. Region III is highly conserved in fungi, but not, apparently,

in plants and animals, while region IV is conserved in all eukaryotes. As expected from such a large protein, it is likely that these regions correspond to the various interactions Pds5 makes with other cohesin subunits.

S. cerevisiae Pds5 mutations that suppress the lethality of *eco1* mutants and are therefore defective in releasing activity cluster in two domains (Rowland et al., 2009): the first cluster is found in and around the conserved APDAP loop (116–120 LtPds5 numbering), as well as the nearby and conserved glutamate E181 (Lt: E210). Mutations in this region either abolish (A88P, Lt: A116) or reduce (E181K, Lt: E210) association of Wapl with chromosomal cohesin in vivo (Chan et al., 2012) and are therefore implicated in binding Wapl (Figure 3A, region II, N-terminal region). The second cluster of *eco1* lethality suppressors is found within the (conserved within fungi) R578, L582, and E602 (Lt: R609, L613, and E633) patch on one side of the extension domain (Figure 3A, region III) (Rowland et al., 2009). Mutations here do not seem to affect Wapl recruitment (Chan et al., 2012) and must affect some other aspect of releasing activity. Pds5 must therefore have a role in releasing activity beyond merely recruiting Wapl.

Implications for Releasing Activity

The Scc1 region that is shown here (Figures 1C and 1D) to bind directly to Pds5 contains a previously unreported motif, conserved in fungi, animals, and plants (Figure S2), which is only 10 to 20 amino acids downstream of the part of Scc1 that has been shown to bind to the coiled-coil segment of Smc3 (Figure 3B, green domain and green arrow) (Gligoris et al., 2014). This observation has two implications: first, the mode by which Scc1 binds Pds5 as elucidated here will prove similar in other eukaryotes. Second, Pds5 may be positioned such that its N-terminal region could lie close to the Smc3's ATPase head and possibly Smc3's K112 and K113, whose acetylation is so crucial for releasing activity. Recent crosslinking experiments support the idea of contacts between Pds5 and Smc3 head and coiled-coil domains (Huis in 't Veld et al., 2014).

Potential Structural Similarity of Pds5, Scc3, and Scc2

When comparing the structure of Pds5 with the other HEAT-repeat-containing subunit of cohesin, Scc3 (Hara et al., 2014; Roig et al., 2014), striking similarities appear: both proteins contain hook, spine, extension, and nose and, intriguingly, bind their corresponding conserved Scc1 sites (Figures S2 and S3) between the spine and the hook, forming a closed ring, and possibly creating another case of topological entrapment in the system (Figure 3C). Furthermore, both Pds5 and Scc3 have similar overall dimensions and separate the N- and C-terminal parts by a large distance. Because the precise amounts of bending at each HEAT repeat are different, overall structural alignments produce poor fits, although Pds5 subdomains containing the canonical HEAT repeat fold can be aligned reasonably well on their counterparts in Scc3/SA2 (Figure S6).

Furthermore, recent single-particle electron microscopy of the cohesin loader subunit Scc2, another HEAT-repeat-containing protein, shows overall architecture similar to that of Scc3 and Pds5 (Chao et al., 2015; Hinshaw et al., 2015). We speculate that these architectural similarities point toward shared mechanisms between Scc3 and Pds5 and, probably, Scc2.

Figure 3B depicts all structurally known cohesin subunits (except Wapl) to scale, showing the large sizes of Pds5 and Scc3 with respect to the Smc ATPase head domains and their attached coiled coils. It is clear that Scc1 plays a key role in the architecture of the complex as its path most likely orchestrates the positions of the various components.

The structures will provide the basis for determining which parts of Pds5 promote Smc acetylation, prevent Smc3 deacetylation, and help to maintain long-term cohesion. In the long-run they will help clarify the mechanism by which cohesin is released from chromosomes.

EXPERIMENTAL PROCEDURES

Full details are provided in the Supplemental Experimental Procedures.

Lachancea thermotolerans Pds5 (XP_002553028.1) was expressed as a C-terminal His_6-tagged fusion in *Escherichia coli* using a T7 plasmid system and purified using metal affinity chromatography, anion exchange, and size-exclusion chromatography. Selenomethionine-labeled LtPds5 proteins were expressed using a published feedback inhibition procedure (van den Ent et al., 1999; Van Duyne et al., 1993) and purified using the same protocol for the native proteins. Scc1 peptide was chemically synthesized as were two otherwise identical, selenomethionine-substituted mutant peptides, Y127SeMet and L128SeMet. After crystallization, Se-Met SAD X-ray diffraction datasets were collected on beamlines i03 at Diamond Light Source and id23eh1 at the ESRF. The apo structure was determined by SAD using established procedures as implemented in the Crank2 pipeline (Skubák and Pannu, 2013). The Scc1 complex structure was determined by molecular replacement using fragments of the apo structure as search models. Modeling of the Scc1 sequence was guided by two additional selenium SAD experiments using peptides containing SeMet residues in two positions: LtScc1(Y127SeMet) and LtScc1(L128SeMet). For refinement, the datasets (Table 1) were corrected for anisotropy using the UCLA Diffraction Anisotropy Server (http://services.mbi.ucla.edu/anisoscale/) (Strong et al., 2006). Co-immunoprecipitations (coIPs) were performed using strains with epitope-tagged yeast strains, expressing proteins from endogenous promoters. IPs used HA epitope-directed commercial antibodies, and PK epitopes were also detected with commercial antibodies. Calibrated ChIP-seq was performed as described (Hu et al., 2015).

ACCESSION NUMBERS

The accession numbers for the coordinates and structure factors reported in this paper have been uploaded to the Protein Data Bank: 5F0N (apo-LtPds5) and 5F0O (LtPds5:LtScc1 peptide complex).

SUPPLEMENTAL INFORMATION

Supplemental Information includes Supplemental Experimental Procedures and six figures and can be found with this article online at http://dx.doi.org/10.1016/j.celrep.2016.02.020.

AUTHOR CONTRIBUTIONS

B.-G.L., M.B.R., N.P., K.N., and J.L. designed and conducted the experiments. B.-G.L. and M.J. performed protein sample preparation. B.-G.L. crystallized and collected X-ray datasets. B.-G.L. and J.L. determined the crystal structures. M.B.R. and J.M. performed mutant viability assays. M.B.R. performed coIP and ChIP-seq, and N.P. analyzed ChIP-seq. B.-G.L., K.N., and J.L. analyzed data and prepared the manuscript.

ACKNOWLEDGMENTS

The authors thank Thomas Gligoris (Oxford) for help with the design of the Scc1 peptide. The authors acknowledge support at beamlines I03, Diamond

Light Source, UK and id23eh1, ESRF, France. This work was funded by the Medical Research Council (U105184326 to J.L.; MR/L018047/1 to J.L. and K.N.), the Wellcome Trust (091859/Z/10/Z to K.N.), and Cancer Research UK (C573/A 12386 to K.N.).

Received: December 1, 2015
Revised: January 12, 2016
Accepted: January 29, 2016
Published: February 25, 2016

REFERENCES

Arumugam, P., Gruber, S., Tanaka, K., Haering, C.H., Mechtler, K., and Nasmyth, K. (2003). ATP hydrolysis is required for cohesin's association with chromosomes. Curr. Biol. *13*, 1941–1953.

Arumugam, P., Nishino, T., Haering, C.H., Gruber, S., and Nasmyth, K. (2006). Cohesin's ATPase activity is stimulated by the C-terminal winged-helix domain of its kleisin subunit. Curr. Biol. *16*, 1998–2008.

Beckouët, F., Hu, B., Roig, M.B., Sutani, T., Komata, M., Uluocak, P., Katis, V.L., Shirahige, K., and Nasmyth, K. (2010). An Smc3 acetylation cycle is essential for establishment of sister chromatid cohesion. Mol. Cell *39*, 689–699.

Borges, V., Lehane, C., Lopez-Serra, L., Flynn, H., Skehel, M., Rolef Ben-Shahar, T., and Uhlmann, F. (2010). Hos1 deacetylates Smc3 to close the cohesin acetylation cycle. Mol. Cell *39*, 677–688.

Chan, K.L., Roig, M.B., Hu, B., Beckouët, F., Metson, J., and Nasmyth, K. (2012). Cohesin's DNA exit gate is distinct from its entrance gate and is regulated by acetylation. Cell *150*, 961–974.

Chan, K.L., Gligoris, T., Upcher, W., Kato, Y., Shirahige, K., Nasmyth, K., and Beckouët, F. (2013). Pds5 promotes and protects cohesin acetylation. Proc. Natl. Acad. Sci. USA *110*, 13020–13025.

Chao, W.C., Murayama, Y., Muñoz, S., Costa, A., Uhlmann, F., and Singleton, M.R. (2015). Structural studies reveal the functional modularity of the Scc2-Scc4 cohesin loader. Cell Rep. *12*, 719–725.

Ciosk, R., Shirayama, M., Shevchenko, A., Tanaka, T., Toth, A., Shevchenko, A., and Nasmyth, K. (2000). Cohesin's binding to chromosomes depends on a separate complex consisting of Scc2 and Scc4 proteins. Mol. Cell *5*, 243–254.

Deardorff, M.A., Bando, M., Nakato, R., Watrin, E., Itoh, T., Minamino, M., Saitoh, K., Komata, M., Katou, Y., Clark, D., et al. (2012). HDAC8 mutations in Cornelia de Lange syndrome affect the cohesin acetylation cycle. Nature *489*, 313–317.

Gandhi, R., Gillespie, P.J., and Hirano, T. (2006). Human Wapl is a cohesin-binding protein that promotes sister-chromatid resolution in mitotic prophase. Curr. Biol. *16*, 2406–2417.

Gligoris, T.G., Scheinost, J.C., Bürmann, F., Petela, N., Chan, K.L., Uluocak, P., Beckouët, F., Gruber, S., Nasmyth, K., and Löwe, J. (2014). Closing the cohesin ring: structure and function of its Smc3-kleisin interface. Science *346*, 963–967.

Gruber, S., Arumugam, P., Katou, Y., Kuglitsch, D., Helmhart, W., Shirahige, K., and Nasmyth, K. (2006). Evidence that loading of cohesin onto chromosomes involves opening of its SMC hinge. Cell *127*, 523–537.

Haering, C.H., Löwe, J., Hochwagen, A., and Nasmyth, K. (2002). Molecular architecture of SMC proteins and the yeast cohesin complex. Mol. Cell *9*, 773–788.

Haering, C.H., Schoffnegger, D., Nishino, T., Helmhart, W., Nasmyth, K., and Löwe, J. (2004). Structure and stability of cohesin's Smc1-kleisin interaction. Mol. Cell *15*, 951–964.

Haering, C.H., Farcas, A.M., Arumugam, P., Metson, J., and Nasmyth, K. (2008). The cohesin ring concatenates sister DNA molecules. Nature *454*, 297–301.

Hara, K., Zheng, G., Qu, Q., Liu, H., Ouyang, Z., Chen, Z., Tomchick, D.R., and Yu, H. (2014). Structure of cohesin subcomplex pinpoints direct shugoshin-Wapl antagonism in centromeric cohesion. Nat. Struct. Mol. Biol. *21*, 864–870.

Hartman, T., Stead, K., Koshland, D., and Guacci, V. (2000). Pds5p is an essential chromosomal protein required for both sister chromatid cohesion and condensation in Saccharomyces cerevisiae. J. Cell Biol. *151*, 613–626.

Hinshaw, S.M., Makrantoni, V., Kerr, A., Marston, A.L., and Harrison, S.C. (2015). Structural evidence for Scc4-dependent localization of cohesin loading. eLife *4*, e06057.

Hu, B., Itoh, T., Mishra, A., Katoh, Y., Chan, K.L., Upcher, W., Godlee, C., Roig, M.B., Shirahige, K., and Nasmyth, K. (2011). ATP hydrolysis is required for relocating cohesin from sites occupied by its Scc2/4 loading complex. Curr. Biol. *21*, 12–24.

Hu, B., Petela, N., Kurze, A., Chan, K.L., Chapard, C., and Nasmyth, K. (2015). Biological chromodynamics: a general method for measuring protein occupancy across the genome by calibrating ChIP-seq. Nucleic Acids Res. *43*, e132.

Huis in 't Veld, P.J., Herzog, F., Ladurner, R., Davidson, I.F., Piric, S., Kreidl, E., Bhaskara, V., Aebersold, R., and Peters, J.M. (2014). Characterization of a DNA exit gate in the human cohesin ring. Science *346*, 968–972.

Ivanov, D., Schleiffer, A., Eisenhaber, F., Mechtler, K., Haering, C.H., and Nasmyth, K. (2002). Eco1 is a novel acetyltransferase that can acetylate proteins involved in cohesion. Curr. Biol. *12*, 323–328.

Kueng, S., Hegemann, B., Peters, B.H., Lipp, J.J., Schleiffer, A., Mechtler, K., and Peters, J.M. (2006). Wapl controls the dynamic association of cohesin with chromatin. Cell *127*, 955–967.

Losada, A., Hirano, M., and Hirano, T. (1998). Identification of Xenopus SMC protein complexes required for sister chromatid cohesion. Genes Dev. *12*, 1986–1997.

Losada, A., Yokochi, T., Kobayashi, R., and Hirano, T. (2000). Identification and characterization of SA/Scc3p subunits in the Xenopus and human cohesin complexes. J. Cell Biol. *150*, 405–416.

Muir, K.W., Kschonsak, M., Li, Y., Metz, J., Haering, C.H., and Panne, P. (2016). Structure of the Pds5-Scc1 complex and implications for cohesin function. Cell Rep. *14*, this issue, 2116–2126.

Nasmyth, K., and Haering, C.H. (2009). Cohesin: its roles and mechanisms. Annu. Rev. Genet. *43*, 525–558.

Nishiyama, T., Ladurner, R., Schmitz, J., Kreidl, E., Schleiffer, A., Bhaskara, V., Bando, M., Shirahige, K., Hyman, A.A., Mechtler, K., and Peters, J.M. (2010). Sororin mediates sister chromatid cohesion by antagonizing Wapl. Cell *143*, 737–749.

Onn, I., Heidinger-Pauli, J.M., Guacci, V., Unal, E., and Koshland, D.E. (2008). Sister chromatid cohesion: a simple concept with a complex reality. Annu. Rev. Cell Dev. Biol. *24*, 105–129.

Panizza, S., Tanaka, T., Hochwagen, A., Eisenhaber, F., and Nasmyth, K. (2000). Pds5 cooperates with cohesin in maintaining sister chromatid cohesion. Curr. Biol. *10*, 1557–1564.

Rankin, S., Ayad, N.G., and Kirschner, M.W. (2005). Sororin, a substrate of the anaphase-promoting complex, is required for sister chromatid cohesion in vertebrates. Mol. Cell *18*, 185–200.

Riedel, C.G., Katis, V.L., Katou, Y., Mori, S., Itoh, T., Helmhart, W., Gálová, M., Petronczki, M., Gregan, J., Cetin, B., et al. (2006). Protein phosphatase 2A protects centromeric sister chromatid cohesion during meiosis I. Nature *441*, 53–61.

Roig, M.B., Löwe, J., Chan, K.L., Beckouët, F., Metson, J., and Nasmyth, K. (2014). Structure and function of cohesin's Scc3/SA regulatory subunit. FEBS Lett. *588*, 3692–3702.

Rolef Ben-Shahar, T., Heeger, S., Lehane, C., East, P., Flynn, H., Skehel, M., and Uhlmann, F. (2008). Eco1-dependent cohesin acetylation during establishment of sister chromatid cohesion. Science *321*, 563–566.

Rowland, B.D., Roig, M.B., Nishino, T., Kurze, A., Uluocak, P., Mishra, A., Beckouët, F., Underwood, P., Metson, J., Imre, R., et al. (2009). Building sister chromatid cohesion: smc3 acetylation counteracts an antiestablishment activity. Mol. Cell *33*, 763–774.

Schmitz, J., Watrin, E., Lénárt, P., Mechtler, K., and Peters, J.M. (2007). Sororin is required for stable binding of cohesin to chromatin and for sister chromatid cohesion in interphase. Curr. Biol. *17*, 630–636.

Skubák, P., and Pannu, N.S. (2013). Automatic protein structure solution from weak X-ray data. Nat. Commun. *4*, 2777.

Strong, M., Sawaya, M.R., Wang, S., Phillips, M., Cascio, D., and Eisenberg, D. (2006). Toward the structural genomics of complexes: crystal structure of a PE/PPE protein complex from Mycobacterium tuberculosis. Proc. Natl. Acad. Sci. USA *103*, 8060–8065.

Sutani, T., Kawaguchi, T., Kanno, R., Itoh, T., and Shirahige, K. (2009). Budding yeast Wpl1(Rad61)-Pds5 complex counteracts sister chromatid cohesion-establishing reaction. Curr. Biol. *19*, 492–497.

Tanaka, K., Hao, Z., Kai, M., and Okayama, H. (2001). Establishment and maintenance of sister chromatid cohesion in fission yeast by a unique mechanism. EMBO J. *20*, 5779–5790.

Tang, Z., Shu, H., Qi, W., Mahmood, N.A., Mumby, M.C., and Yu, H. (2006). PP2A is required for centromeric localization of Sgo1 and proper chromosome segregation. Dev. Cell *10*, 575–585.

Tong, K., and Skibbens, R.V. (2014). Cohesin without cohesion: a novel role for Pds5 in Saccharomyces cerevisiae. PLoS ONE *9*, e100470.

Tóth, A., Ciosk, R., Uhlmann, F., Galova, M., Schleiffer, A., and Nasmyth, K. (1999). Yeast cohesin complex requires a conserved protein, Eco1p(Ctf7), to establish cohesion between sister chromatids during DNA replication. Genes Dev. *13*, 320–333.

Uhlmann, F., Lottspeich, F., and Nasmyth, K. (1999). Sister-chromatid separation at anaphase onset is promoted by cleavage of the cohesin subunit Scc1. Nature *400*, 37–42.

Unal, E., Heidinger-Pauli, J.M., Kim, W., Guacci, V., Onn, I., Gygi, S.P., and Koshland, D.E. (2008). A molecular determinant for the establishment of sister chromatid cohesion. Science *321*, 566–569.

van den Ent, F., Lockhart, A., Kendrick-Jones, J., and Löwe, J. (1999). Crystal structure of the N-terminal domain of MukB: a protein involved in chromosome partitioning. Structure *7*, 1181–1187.

Van Duyne, G.D., Standaert, R.F., Karplus, P.A., Schreiber, S.L., and Clardy, J. (1993). Atomic structures of the human immunophilin FKBP-12 complexes with FK506 and rapamycin. J. Mol. Biol. *229*, 105–124.

Vaur, S., Feytout, A., Vazquez, S., and Javerzat, J.P. (2012). Pds5 promotes cohesin acetylation and stable cohesin-chromosome interaction. EMBO Rep. *13*, 645–652.

Waizenegger, I.C., Hauf, S., Meinke, A., and Peters, J.M. (2000). Two distinct pathways remove mammalian cohesin from chromosome arms in prophase and from centromeres in anaphase. Cell *103*, 399–410.

Weitzer, S., Lehane, C., and Uhlmann, F. (2003). A model for ATP hydrolysis-dependent binding of cohesin to DNA. Curr. Biol. *13*, 1930–1940.

Xiong, B., Lu, S., and Gerton, J.L. (2010). Hos1 is a lysine deacetylase for the Smc3 subunit of cohesin. Curr. Biol. *20*, 1660–1665.

Cell Reports, Volume *14*

Supplemental Information

Crystal Structure of the Cohesin Gatekeeper Pds5 and in Complex with Kleisin Scc1

Byung-Gil Lee, Maurici B. Roig, Marijke Jansma, Naomi Petela, Jean Metson, Kim Nasmyth, and Jan Löwe

Inventory of Supplemental Information

Supplemental Figure S1

Electron density map supporting the main finding of the paper, the crystal structure of Pds5 (belongs to Figure 1A).

Supplemental Figures S2 & S3

Multiple sequence alignments showing the conservation of Scc1 binding to Pds5 and Scc3 (belongs to Figure 1C).

Supplemental Figure S4

Lethality analysis of Pds5 and Scc1 mutants, validating the structure of the complex of Pds5 and Scc1 (belongs to Figure 2A &B).

Supplemental Figure S5

Control experiments supporting the ChIP-seq experiment in Figure 2C.

Supplemental Figure S6

Superposition of parts of the Pds5 and previous Scc3 structures, supporting Figure 3C.

Supplemental Experimental Procedures

List of Strains

Supplemental References

Supplementary Figure S1. 2Fo-Fc electron density map of apo Pds5 at 3.2 Å resolution. Related to Figure 1A. The map was contoured at 1.2 σ level. Inset: close up of a section of the spine, showing approximately residues 370-470. The electron density map was of excellent quality except the very N- and C-terminal sections, most likely through disorder in the crystals.

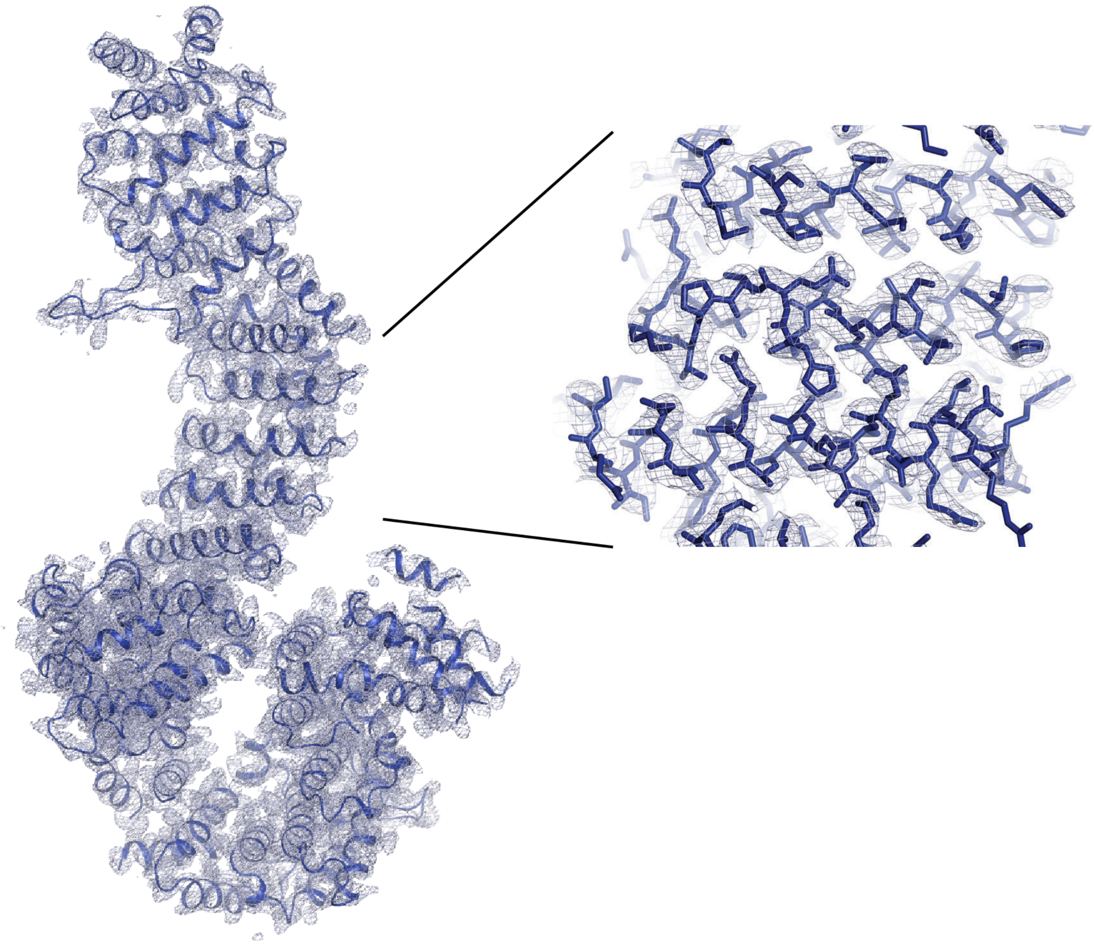

Supplementary Figure S2. Multiple sequence alignment showing conserved Pds5 binding regions within Scc1 kleisins. Related to Figure 1C. Up to 10 sequences similar to each *L. thermotolerans*, *S. cerevisiae*, *S. pombe*, *A. thaliana*, *H. sapiens* and *D. melanogaster* Scc1 were selected by BLAST (each block evenly sampled down to around 30 % sequence identity) and sequences were aligned by Clustal Omega. Resides 120-141 are shown for the Pds5 binding site in Scc1, containing more than the peptide co-crystallised here with Pds5 (*Lt* numbering; *Lt*Scc1 is the first sequence in the alignment.). LtPds5 residues 125-141 were built in the co-crystal structure (Table 1, Figure 1C, D).

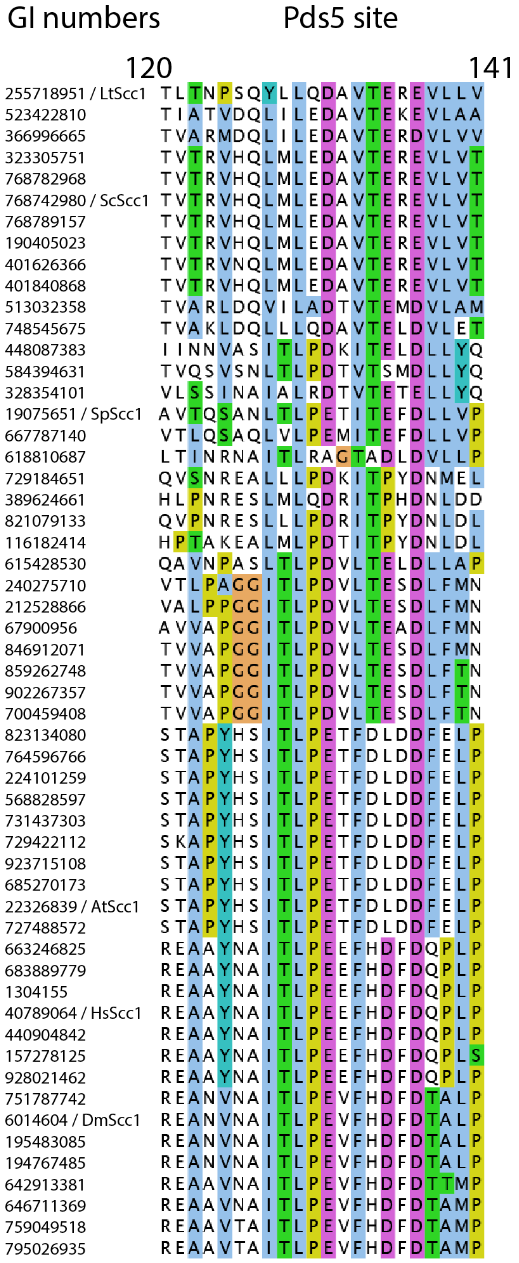

Supplementary Figure S3. Multiple sequence alignment showing conserved Scc3 binding regions within Scc1 kleisins from humans to yeast. Related to Figures 1C and 3C. 500 sequences similar to human and yeast Scc1 were selected by BLAST and all sequences (including from *L. thermotolerans*) were aligned by Clustal Omega. The alignment was truncated to 60 sequences without re-aligning. Resides 326-400 are shown for the Scc3 binding site in Scc1 corresponding to the fragment co-crystallised with SA2/Scc3 previously (*Lt* numbering; *Lt*Scc1 is the last sequence in the alignment.). Note that helix B is most likely missing from the Scc1 fragment in yeasts and related organisms but otherwise the alignment indicates that the binding mode of Scc1 to Scc3 is conserved.

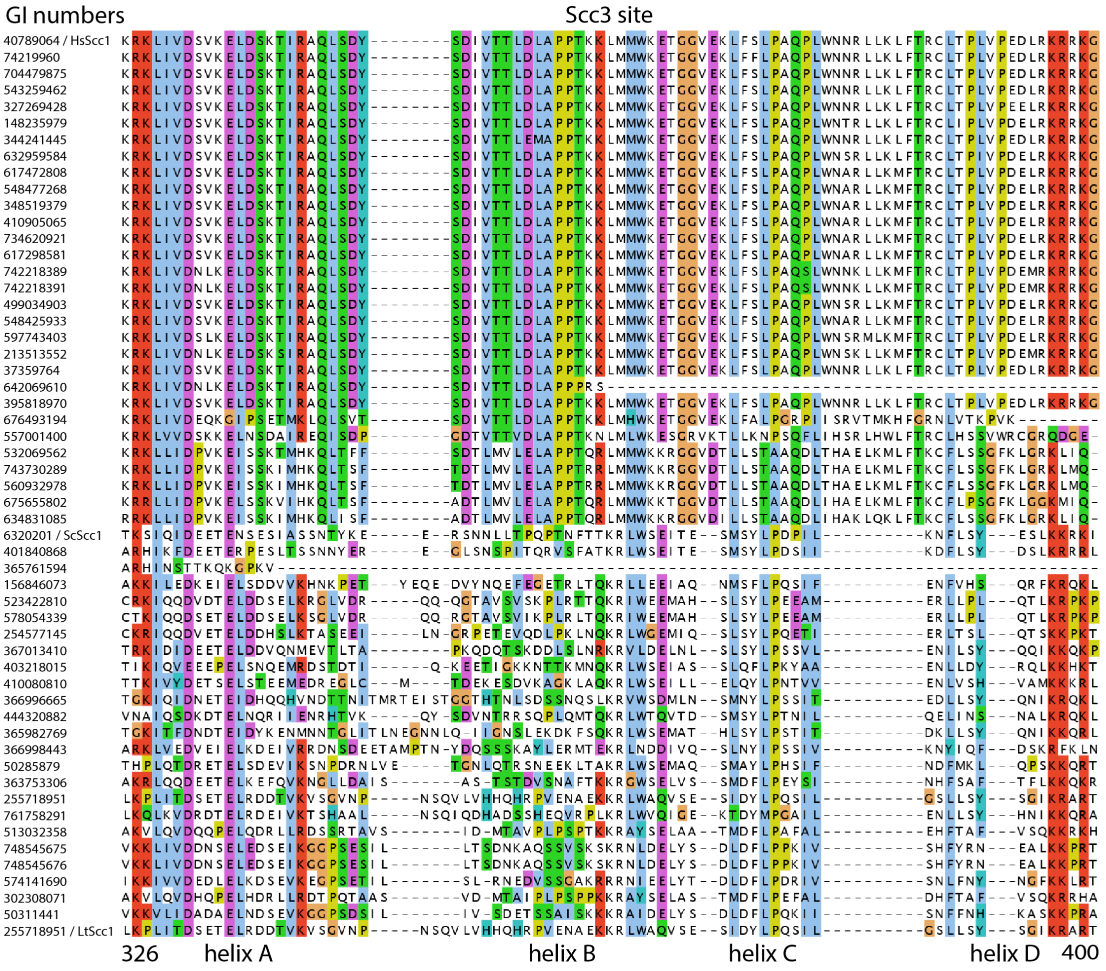

Supplementary Figure S4. ***S. cerevisiae*** **Pds5(Y458K) growth tests.** Related to Figures 2A & B. Wild type cells (K699, see list of strains) and cells with the endogenous *PDS5* locus deleted and expressing *PDS5* WT (K25118) or *pds5* (Y458K) (K25126) integrated at the *lys2* locus were streaked on YEPD plates and incubated at different temperatures. Note that ScPds5 Y458 corresponds to LtPds5 Y493 (labelled in Figure 1D).

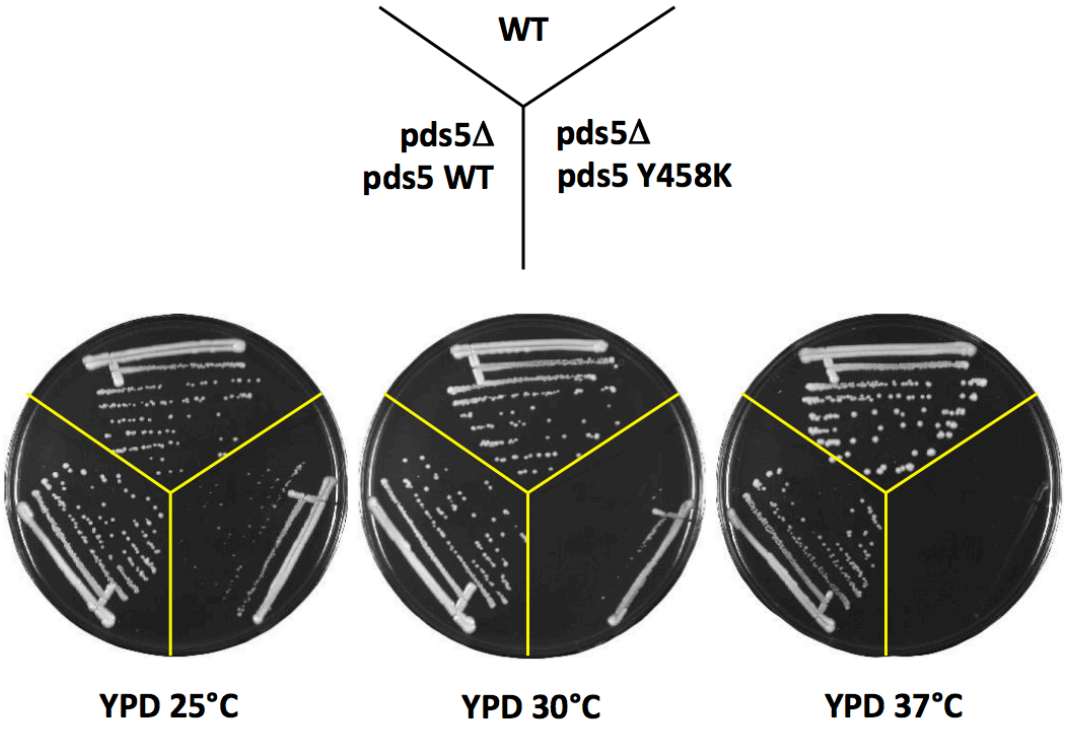

Supplementary Figure S5. Reduced occupancy of pds5(Y458K) across the genome. Related to Figure 2C. (A) Calibrated ChIP-seq profiles of Pds5 (strain K25120) and pds5(Y458K) (K25128) showing the number of reads at each base pair on chromosome I. (B) Calibrated ChIP-seq profiles showing the percentage of reads of pds5(Y458K) (K25128) at each base pair away from the CDEIII element, averaged over all sixteen chromosomes with respect to Pds5 (K25120). (C) FACS data showing cycling of the cells used in this figure and Figure 2C (strains K699, K25120, K25128).

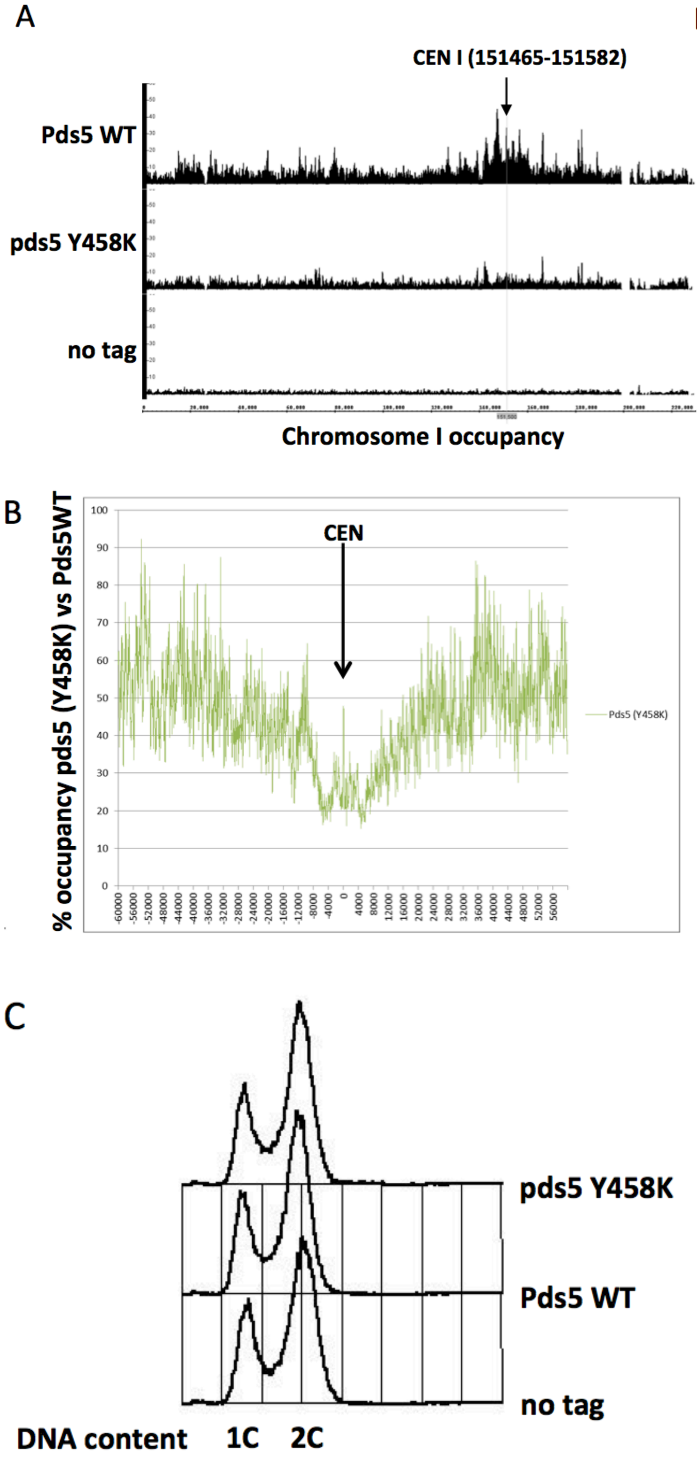

Supplementary Figure S6. Superposition of LtPds5 domains on previously determined Scc3 and SA2 structures. Related to Figure 3C. The LtPds5 structure was divided into four domains (left) that were independently aligned in 3D (PyMOL 1.7.6.2 'cealign' and 'align' commands) against the entire Scc3 (PDB 4UVK, middle) and SA2 (PDB 4PJU, right) structures. No manual adjustments were made. Two parts of Pds5 align reasonably well against their corresponding parts in both Scc3 and SA2. The C-terminal domain aligns less well and does not superimpose at all close to the C-terminus. It should be noted that the aligning parts show the highest conservation of the canonical HEAT repeat fold.

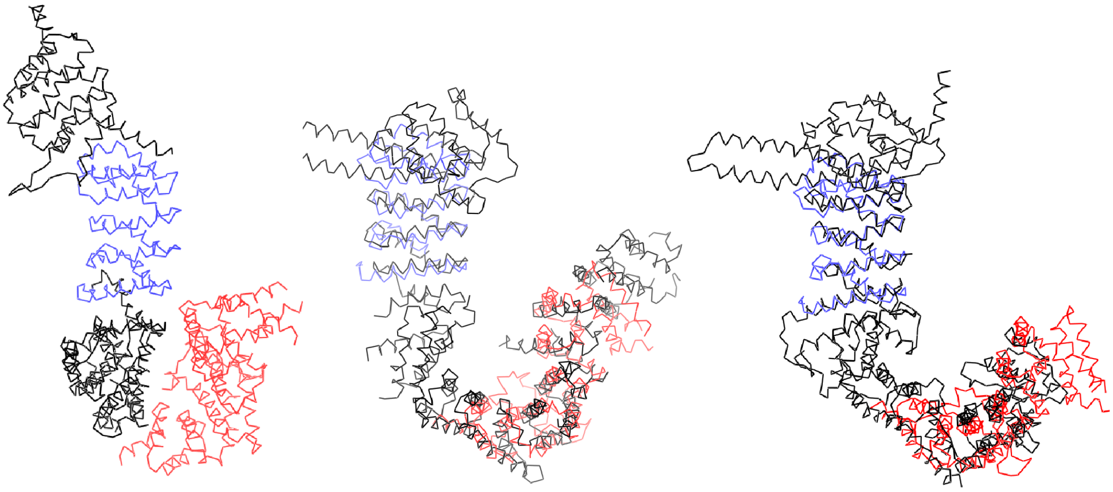

**Pds5**	**SA2**	**Scc3**
PDB 5F0O	PDB 4PJU	PDB 4UVK
N-terminal: 76-278	-	-
middle 1: 288-470	RMSD 3.3 Å	RMSD 3.4 Å
middle 2: 471-726	-	-
C-terminal: 763-1109	RMSD 5.1 Å	RMSD 5.5 Å

Supplemental Experimental Procedures

Cloning, overexpression and purification

Lachancea thermotolerans CBS6340 Pds5 (LtPds5, NCBI database identifier XP_002553028.1) was expressed in *E. coli* using a codon-optimised synthetic gene (Epoch Lifescience, TX). Two constructs were used, LtPds5-1, amino acids 35-1221, approx.. 137 kDa and LtPds5-2, amino acids 45-1221, approx. 135 kDa (see Table 1). These were cloned into expression vector pHis17 using Gibson assembly (New England Biolabs, MA), adding the affinity purification tag LHHHHHH at the C-termini. For overexpression, C41(DE3) cells (Lucigen, WI) were transformed with the resulting vectors and grown in 2xTY media and induced with 1 mM IPTG at 16˚C overnight. Cells were harvested and re-suspended in lysis buffer (50 mM Tris-HCl, 150 mM NaCl, 2 mM TCEP and 5% (w/v) glycerol, pH 8.0), and lysed through a Constant Systems cell disruptor at 25 kPSI in the presence of DNase, RNase and EDTA-free protease inhibitors (Roche). The cell lysates were clarified by ultracentrifugation at 200,000 g in a Beckman 45 Ti rotor and applied to nickel resin (5 ml HisTrap HP, GE Healthcare) and eluted with 50-150 mM imidazole in lysis buffer. Fractions containing Pds5 proteins were further purified using anion exchange chromatography (5 ml HiTrap Q FF, GE Healthcare) with gradients of 100-1000 mM NaCl in buffer containing 50 mM Tris-HCl, 100 mM NaCl, 2 mM TCEP, 5 % (w/v) glycerol, pH 8.5. Proteins were concentrated using spin concentrators (Vivaspin, Satorius, 50 kDa MWCO) and further purified using size exclusion chromatography (Sephacryl S300 16/60, GE Healthcare) in buffer containing 50 mM Tris-HCl, 250 mM NaCl, 5 mM TCEP, pH 7.5. Selenomethionine-labeled LtPds5 proteins were expressed using a published feedback inhibition procedure (van den Ent et al., 1999; Van Duyne et al., 1993) and purified using the same protocol for the native proteins. All purifications were performed at 4˚C. The LtScc1 (NCBI database identifier XP_002555756.1) peptide (residues 121-143: LTNPSQYLLQDAVTEREVLLVPG) design was informed by published results (Chan et al., 2013). Two otherwise identical, selenomethionine-substituted mutant peptides, Y127SeMet and L128SeMet, were used to confirm the

orientation of Scc1 polypeptide. All peptides were chemically synthesised (Generon, UK, Cambridge Peptides, UK, and Genscript, USA/Hong Kong).

Crystallisation and data collection

Initial crystallisation experiments were carried out using sitting-drop vapour diffusion with LMB’s in-house high-throughput crystallisation facility at 100 nl volumes (Stock et al., 2005). LtPds5-2 protein (amino acids 45-1221) was crystallised with reservoir solutions containing 100 mM HEPES pH 7.5, and 1.3-1.6 M lithium sulphate, and both native and selenomethionine-substituted crystals were obtained in similar conditions. Diffraction quality crystals were grown at 20 ˚C using concentrations of 10 mg/ml, mixed with reservoir solution 0.3 - 0.4 times the protein solution's volume at 1.2 µl volumes. Crystals were observed after 2-3 weeks and were cryo-protected by serially increasing lithium sulphate concentration up to 2.2 M in the drop and subsequent flash freezing in liquid nitrogen. For Pds5-Scc1 co-crystallization, LtPds5-1 protein (amino acids 35-1221) at 8 mg/ml was mixed with LtScc1 peptide (amino acids 121-143) in five times molar excess. Complex crystals were obtained with reservoir solution containing 50 mM sodium cacodylate pH 6.5, 1.4 – 1.6 M ammonium sulphate and 5 mM magnesium acetate, and grew within a week. The crystals were cryo-protected by transferring to reservoir solution supplemented with 1.2 M sodium malonate and flash frozen in liquid nitrogen before data collection. Co-crystals of Pds5 and two selenomethionine-substituted Scc1 mutant peptides (Y127SeMet and L128SeMet) were grown with LtPds5-2 protein under reservoir solution containing 50 mM sodium cacodylate pH 6.5, 1.4 M lithium sulphate, 40 mM sodium citrate. The complex crystals were cryo-protected by serially increasing lithium sulphate in the drop and flash frozen in liquid nitrogen. Diffraction data were collected at 100 K on beamlines i03 at Diamond Light Source (Harwell, UK) and id23eh1 at the ESRF (Grenoble, France).

Structure determination

Diffraction data were integrated and scaled with XDS (Kabsch, 2010) and SCALA (Winn et al., 2011). Phasing was done by SeMet SAD combining data from two separate crystals in order to increase multiplicity and anomalous signal.

Selenium positions were identified and SAD phases were calculated using SHELXC/D/E (Sheldrick, 2008) and PHASER (McCoy et al., 2007). An initial atomic model was obtained using Crank2 (Skubak and Pannu, 2013), and manually improved using COOT (Emsley et al., 2010) and MAIN (Turk, 2013). For refinement, the high resolution native apo dataset (Table 1) was corrected for anisotropy using the UCLA Diffraction Anisotropy Server (services.mbi.ucla.edu/anisoscale/) (Strong et al., 2006), and the model was further rebuilt and refined in cycles at 3.2 Å resolution, manually rebuilt as above and refined with REFMAC (Murshudov et al., 1997) and PHENIX (Adams et al., 2010). The *Lt*Pds5-*Lt*Scc1 complex dataset was even more anisotropic and was also corrected using the UCLA server. Data extended to 3.5 Å in two directions (Table 1) and 4.5 in the third, leading to an estimate of overall resolution of 3.6 Å. Note that dataset statistics listed in Table 1 are those of the uncorrected data at 3.6 Å resolution before applying anisotropy correction. The complex structure was solved by molecular replacement with PHASER. Due to overall conformational changes between apo-Pds5 and the Pds5-Scc1 complex structures, molecular replacement was performed with the apo structure cut into 4 roughly equal sized subdomains. After improving the atomic model of Pds5 within the complex structure by manual building and refinement as described above, strong extra electron density was located and the LtScc1 peptide was fitted. Modelling of the Scc1 sequence at this low resolution was guided by two additional selenium SAD experiments (details of data not shown) using peptides containing SeMet residues in two positions: LtScc1(Y127SeMet) and LtScc1(L128SeMet). Phases were calculated using ANODE (Thorn and Sheldrick, 2011) and the refined Pds5 structure in the complex crystals. The entire model was then built and refined in cycles as for the apo structure. For R-factors and other statistics of the data and models, please refer to Table 1. Because of low resolution, no waters or ions were added to any of the structures. Figures were prepared using PYMOL (Schroedinger). Coordinates and structure factors were deposited in the Protein Data Bank (PDB) with accession numbers 5F0N (apo-LtPds5) and 5F0O (LtPds5:LtScc1 peptide complex).

Cell viability analysis of S. cerevisiae Pds5 and Scc1 mutants

The corresponding residues in *S. cerevisiae* Pds5 and Scc1 were located by sequence alignments. Mutant versions of Pds5 (under its native promoter) were incorporated at the *lys2* locus in heterozygous *PDS5/pds5Δ* diploid cells (K25105, see List of Strains in Supplementary Data). Diploids were sporulated on SpoVB media plates and tetrads dissected at 25°C on YPD media plates. The genotype of the resulting haploids was determined by replica plating, and viable cells with only the ectopic copy were additionally tested for temperature sensitivity streaking cells on YEP glucose plates at 25°C, 30°C and 37°C (Figure S4). Mutant versions of *scc1* (under its native promoter) were incorporated at the *leu2* locus in heterozygous *SCC1/scc1D* diploid cells (K12714) and analysed in a similar manner. All mutations were confirmed by DNA sequencing.

Co-immunoprecipitations

Strains were grown in YEPD at 25°C to OD_{600nm} = 0.7 and 70 OD units were washed in ice-cold PBS and frozen at -80°C. Pellets were thawed and re-suspended in lysis buffer (50 mM Tris/HCl, 100 mM NaCl, 5 mM EDTA, 1 mM DTT, 1 mM PMSF, Roche Complete Protease Inhibitors) and lysed in a FastPrep-24 (MP Biomedicals) disruptor 3 times for 1 min at 5.5 m/s with an equal volume of acid-washed glass beads (Sigma). Lysates were cleared by centrifugation at 13 Krpm for 30 min at 4°C and the protein amount of the supernatant was quantified with a Bradford assay. For cohesion immuneprecipitation, 150 µl of washed anti-HA High Affinity Matrix (Roche) was added to the cleared lysates and incubated over night at 4°C while rotating. The incubated anti-HA High Affinity Matrix was washed 3 times with 1 ml of lysis buffer, re-suspended in 60 µl of SDS-PAGE sample buffer and incubated at 95 °C for 10 min. 10 µl of each sample was loaded onto a precast Tris-acetate gel (3-8%, NuPAGE), followed by Western blotting and immunodetection of the PK epitopes with anti-PK antibody (AbD Serotec) and the HA epitopes with anti HA antibody (12CA5).

Calibrated ChIP-seq analysis

Calibrated ChIP-seq was performed as described (Hu et al., 2015) using K23308, K699, K25120 and K25128 strains for this assay.

Pds5 sequence conservation mapping on Pds5 crystal structure

500 sequences most similar to *L. thermotolerans* Pds5 were selected from a BLAST search and aligned using Clustal Omega (http://clustal.org), before mapping sequence conservation at each residue position onto the structure with ConSurf (http://consurf.tau.ac.il) (Ashkenazy et al., 2010).

List of Strains:

All yeast strains are derivatives of W303, except K23308.

K699 *MATa, ade2-1, trp1-1, can1-100, leu2-3,112,his3-11,15, ura3, GAL, psi+*

K12714 *MATa/alpha scc1:KanMx / WT*

K23308 *C.glabrata, MATa, Scc1PK9::NatMX*

K24593 *MATalpha, scc1:KanMX, Scc1-HA6 in pRS305H*

K24595 *MATalpha, leu2:Scc1-HA6:leu2*

K24958 *MATa/alpha, scc1:KanMX / WT, leu2:Scc1(V137K)-HA6:leu2*

K25002 *MATa, leu2:Scc1(V137K)-HA6:leu2*

K25105 *MATa/alpha pds5::HIS / WT*

K25106 *MATa/alpha, pds5::HIS / WT, lys2:Pds5-PK9-HphMX:lys2*

K25108 *MATa/alpha, pds5::HIS / WT, lys2:Pds5(Y458K)-PK9-HphMX:lys2*

K25118 *MATa pds5::HIS, lys2:Pds5-PK9-HphMX:lys2*

K25120 *MATa, lys2:Pds5-PK9-HphMX:lys2*

K25126 *MATa pds5::HIS, lys2:Pds5(Y458K)-PK9-HphMX:lys2*

K25128 *MATa, lys2:Pds5(Y458K)-PK9-HphMX:lys2*

K25166 *MATa/alpha scc1:KanMX / WT, leu2:Scc1-HA6:leu2*

K25202 *MATa, pds5::HIS, lys2:Pds5-PK9-HphMX:lys2, leu2:Scc1-HA6:leu2*

K25204 *MATalpha, lys2:Pds5-PK9-HphMX:lys2, scc1:KanMX, leu2:Scc1-HA6:leu2*

K25206 *MATalpha, pds5::HIS, lys2:Pds5-PK9-HphMX:lys2, leu2:Scc1(V137K)-HA6:leu2*

K25210 *MATalpha, lys2:Pds5(Y458K)-PK9-HphMX:lys2, scc1:KanMX, leu2:Scc1-HA6:leu2*

Supplemental References

Adams, P. D., Afonine, P. V., Bunkoczi, G., Chen, V. B., Davis, I. W., Echols, N., Headd, J. J., Hung, L. W., Kapral, G. J., Grosse-Kunstleve, R. W., McCoy, A. J., Moriarty, N. W., Oeffner, R., Read, R. J., Richardson, D. C., Richardson, J. S., Terwilliger, T. C., and Zwart, P. H. (2010). PHENIX: a comprehensive Python-based system for macromolecular structure solution. Acta Crystallogr D Biol Crystallogr *66*, 213-221.

Ashkenazy, H., Erez, E., Martz, E., Pupko, T., and Ben-Tal, N. (2010). ConSurf 2010: calculating evolutionary conservation in sequence and structure of proteins and nucleic acids. Nucleic Acids Res. *38*, W529-W533.

Chan, K. L., Gligoris, T., Upcher, W., Kato, Y., Shirahige, K., Nasmyth, K., and Beckouet, F. (2013). Pds5 promotes and protects cohesin acetylation. Proc. Natl. Acad. Sci. U. S. A. *110*, 13020-13025.

Emsley, P., Lohkamp, B., Scott, W. G., and Cowtan, K. (2010). Features and development of Coot. Acta Crystallogr D Biol Crystallogr *66*, 486-501.

Hu, B., Petela, N., Kurze, A., Chan, K. L., Chapard, C., and Nasmyth, K. (2015). Biological chromodynamics: a general method for measuring protein occupancy across the genome by calibrating ChIP-seq. Nucleic Acids Res. *43*, e132.

Kabsch, W. (2010). XDS. Acta Crystallogr D Biol Crystallogr *66*, 125-132.

McCoy, A. J., Grosse-Kunstleve, R. W., Adams, P. D., Winn, M. D., Storoni, L. C., and Read, R. J. (2007). Phaser crystallographic software. J. Appl. Crystallogr. *40*, 658-674.

Murshudov, G. N., Vagin, A. A., and Dodson, E. J. (1997). Refinement of macromolecular structures by the maximum-likelihood method. Acta Crystallogr D Biol Crystallogr *53*, 240-255.

Sheldrick, G. M. (2008). A short history of SHELX. Acta Crystallogr A *64*, 112-122.

Skubak, P., and Pannu, N. S. (2013). Automatic protein structure solution from weak X-ray data. Nat Commun *4*, 2777.

Stock, D., Perisic, O., and Löwe, J. (2005). Robotic nanolitre protein crystallisation at the MRC Laboratory of Molecular Biology. Prog Biophys Mol Biol *88*, 311-327.

Strong, M., Sawaya, M. R., Wang, S., Phillips, M., Cascio, D., and Eisenberg, D. (2006). Toward the structural genomics of complexes: crystal structure of a PE/PPE protein complex from Mycobacterium tuberculosis. Proc. Natl. Acad. Sci. U. S. A. *103*, 8060-8065.

Thorn, A., and Sheldrick, G. M. (2011). ANODE: anomalous and heavy-atom density calculation. J. Appl. Crystallogr. *44*, 1285-1287.

Turk, D. (2013). MAIN software for density averaging, model building, structure refinement and validation. Acta Crystallogr D Biol Crystallogr *69*, 1342-1357.

van den Ent, F., Lockhart, A., Kendrick-Jones, J., and Löwe, J. (1999). Crystal structure of the N-terminal domain of MukB: a protein involved in chromosome partitioning. Structure *7*, 1181-1187.

Van Duyne, G. D., Standaert, R. F., Karplus, P. A., Schreiber, S. L., and Clardy, J. (1993). Atomic structures of the human immunophilin FKBP-12 complexes with FK506 and rapamycin. J. Mol. Biol. *229*, 105-124.

Winn, M. D., Ballard, C. C., Cowtan, K. D., Dodson, E. J., Emsley, P., Evans, P. R., Keegan, R. M., Krissinel, E. B., Leslie, A. G., McCoy, A., McNicholas, S. J., Murshudov, G. N., Pannu, N. S., Potterton, E. A., Powell, H. R., Read, R. J., Vagin, A., and Wilson, K. S. (2011). Overview of the CCP4 suite and current developments. Acta Crystallogr D Biol Crystallogr *67*, 235-242.
